# FOXK2 promotes ovarian cancer stemness by regulating the unfolded protein response pathway

**DOI:** 10.1172/JCI151591

**Published:** 2022-05-16

**Authors:** Yaqi Zhang, Yinu Wang, Guangyuan Zhao, Edward J. Tanner, Mazhar Adli, Daniela Matei

**Affiliations:** 1 Department of Obstetrics and Gynecology,; 2 Driskill Graduate Training Program in Life Sciences, and; 3 Robert H. Lurie Comprehensive Cancer Center, Feinberg School of Medicine, Northwestern University, Chicago, Illinois, USA.; 4 Jesse Brown VA Medical Center, Chicago, Illinois, USA.

**Keywords:** Cell Biology, Oncology, Cancer, Cell stress, Transcription

## Abstract

Understanding the regulatory programs enabling cancer stem cells (CSCs) to self-renew and drive tumorigenicity could identify new treatments. Through comparative chromatin-state and gene expression analyses in ovarian CSCs versus non-CSCs, we identified FOXK2 as a highly expressed stemness-specific transcription factor in ovarian cancer. Its genetic depletion diminished stemness features and reduced tumor initiation capacity. Our mechanistic studies highlight that FOXK2 directly regulated IRE1**α** (encoded by *ERN1*) expression, a key sensor for the unfolded protein response (UPR). Chromatin immunoprecipitation and sequencing revealed that FOXK2 bound to an intronic regulatory element of *ERN1*. Blocking FOXK2 from binding to this enhancer by using a catalytically inactive CRISPR/Cas9 (dCas9) diminished IRE1**α** transcription. At the molecular level, FOXK2-driven upregulation of IRE1**α** led to alternative *XBP1* splicing and activation of stemness pathways, while genetic or pharmacological blockade of this sensor of the UPR inhibited ovarian CSCs. Collectively, these data establish what we believe is a new function for FOXK2 as a key transcriptional regulator of CSCs and a mediator of the UPR, providing insight into potentially targetable new pathways in CSCs.

## Graphical abstract

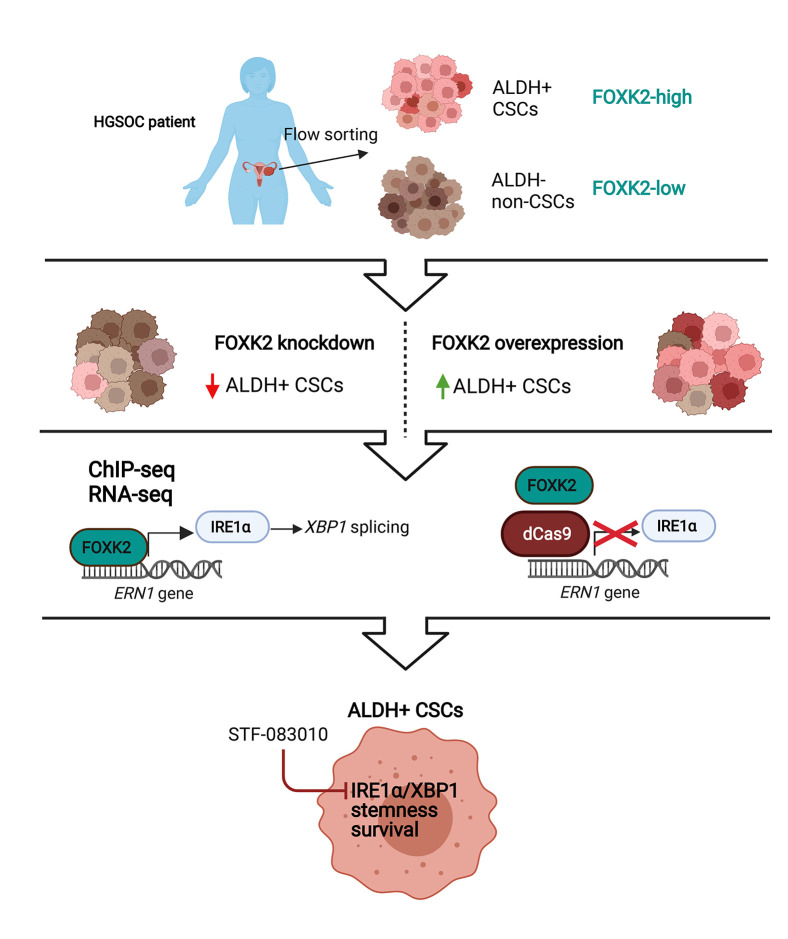

## Introduction

Cancer stem cells (CSCs) represent a small fraction of cells within tumors with self-renewal, differentiation, and tumor-initiation capacity (TIC). CSCs express high levels of stemness-associated transcription factors (TFs) SOX2, NANOG, and OCT4, grow as spheres, generate tumors when injected in small numbers in NOD/SCID mice (1, 2), and have been implicated in tumorigenesis, tumor heterogeneity, and resistance to traditional cytotoxics (1, 3). Although controversy around optimal stemness markers persist, cells with high aldehyde dehydrogenase (ALDH) activity have been shown to possess self-renewal and tumorigenic ability, effectively form spheres, express stemness-associated TFs, and to be quiescent (4–7), thus recapitulating the CSC phenotype across many cancers. In an ovarian cancer (OC) context, we and others have shown that high-ALDH-expressing cancer cells have CSC features and harbor a drug-resistant phenotype (5, 8–10). Apart from ALDH, expression of CD133 has also been recognized as a marker for CSCs in solid tumors, including in OC (11). There is high interest in defining transcriptional drivers of stemness, which could lead to new targetable mechanisms regulating CSCs’ survival and tumorigenicity.

FOXK2 is a member of the forkhead box (FOX) family, which includes several TFs involved in cell metabolism, differentiation, and proliferation (12). Unlike other FOX family TFs, the functions of FOXK2 in cancer are less well understood, although its context-dependent and tumor-specific functions have been recently reported. For example, FOXK2 was shown to promote tumor progression by interacting with Dishevelled (DVL) and activating Wnt signaling in colon cancer models (13), but suppressed estrogen receptor–positive breast cancer cell proliferation by interacting with transcriptional corepressor complexes (14). FOXK2 was also linked to regulation of glycolysis and autophagy (15, 16) and to SOX9-mediated cell proliferation (17). The cellular functions and direct targets of FOXK2 in CSCs and in OC have not been reported to the best of our knowledge.

Regulation of the unfolded protein response (UPR), which is an evolutionarily conserved pathway activated under conditions of cellular stress, is initiated by the sensor protein inositol-requiring enzyme 1α (IRE1α), encoded by the *ERN1* gene, in response to accumulation of unfolded proteins in the lumen of the endoplasmic reticulum (ER) (18). IRE1α has both endoribonuclease and kinase activity, being involved in the splicing of the X-box-binding protein 1 (*XBP1*) mRNA that leads to a spliced form (XBP1s) with potent transcriptional activity (19). The IRE1α/XBP1 pathway was shown to promote breast cancer progression and tumor initiation by activating the hypoxia pathway governed by HIF-1α (20). Even in the absence of obvious cellular stress, HIF-1α and XBP1 were upregulated in cancer cells, indicating that cancer cells are under continuous metabolic stress at baseline, likely due to increased levels of protein production, higher oxidative stress, lower concentrations of nutrients, and hypoxia (21). Although the role of the UPR in cancer has been reported, it is not clearly understood whether the UPR is distinctly regulated in CSCs compared with non-CSCs, and its association with the stemness properties of CSCs is relatively unknown.

By using the assay for transposase-accessible chromatin with high-throughput sequencing (ATAC-seq) we identified *FOXK2* as an actively transcribed gene in ovarian CSCs. Here we show that the expression level of this TF was upregulated in ovarian CSCs compared with non-CSCs and robustly associated with stemness characteristics in vitro and in vivo. Chromatin immunoprecipitation and sequencing (ChIP-seq) identified the UPR stress sensor IRE1α as a direct and previously unrecognized target of FOXK2 in OC cells and tumors. IRE1α inhibition or XBP1 knockdown potently blocked stemness characteristics. Our data establish a previously unappreciated role of FOXK2 in regulating cancer stemness through fine-tuning the intracellular stress defense mechanism governed by IRE1α/XBP1.

## Results

### FOXK2 expression is upregulated in OC cells and enriched in ALDH^+^ CSCs.

**Figure 1:**
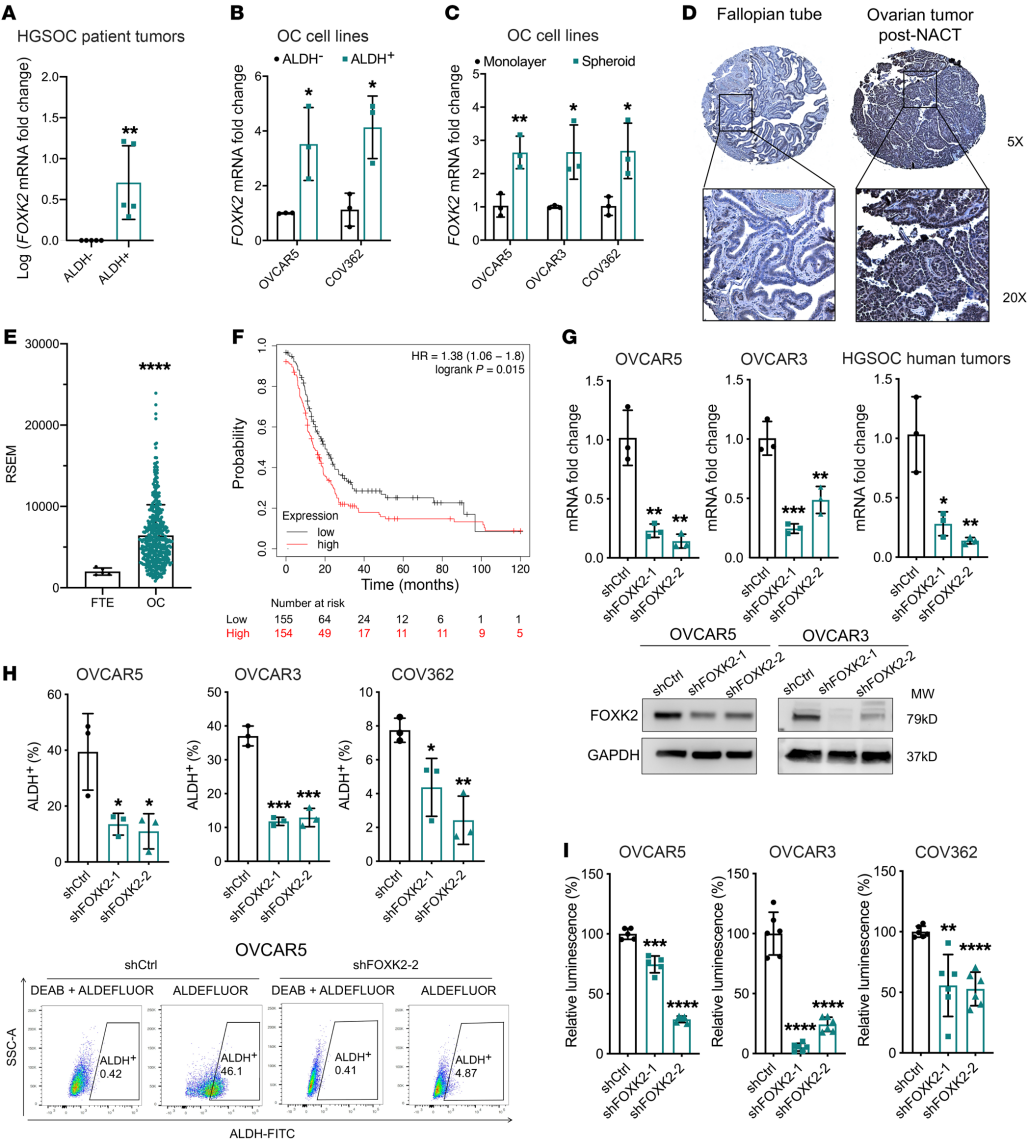
FOXK2 expression is upregulated in ovarian CSCs. (**A** and **B**) *FOXK2* mRNA expression levels measured by qRT-PCR in ALDH^+^ and ALDH^–^ cells sorted by FACS from HGSOC tumors (*n =* 5) (**A**), or from OVCAR5 (*n =* 3) and COV362 (*n =* 3) OC cell lines (**B**). (**C**) *FOXK2* mRNA expression in ALDH-enriched spheroids and monolayers generated from OVCAR5, OVCAR3, and COV362 (*n =* 3 per cell line). (**D**) FOXK2 IHC staining in sections of fallopian tube epithelium (FTE, *n =* 6) and tumors after neoadjuvant chemotherapy (NACT, *n =* 19) from a tissue microarray (TMA). (**E**) FOXK2 expression from RNA-seq data analyzed with RSEM in normal FTE tissue (*n =* 5) and OC tissue (OV, *n =* 427) from TCGA and GTEx databases. (**F**) A Kaplan-Meier plot shows survival of OC patients with high (top 25th percentile, *n =* 155) and low (bottom 25th percentile, *n =* 154) *FOXK2* mRNA expression levels obtained from TCGA and GEO databases (*n =* 614). (**G**) Upper: *FOXK2* expression levels measured by qRT-PCR (*n =* 3) in OVCAR5, OVCAR3, and patient HGSOC primary cells transduced with 2 different shRNAs targeting *FOXK2* (shFOXK2-1 and shFOXK2-2) or control shRNAs (shCtrl). Lower: Western blot of FOXK2 protein levels in shCtrl and shFOXK2 OVCAR5 and OVCAR3 cells. (**H**) Percentage of ALDH^+^ cells determined by flow cytometry analysis in shFOXK2- and shCtrl-transduced OVCAR5, OVCAR3, and COV362 cells (*n =* 3 per cell line) (upper), and representative analysis of the ALDH^+^ cell populations in OVCAR5 cells (lower). (**I**) Relative cell viability in spheroids generated by shFOXK2 and shCtrl OVCAR5, OVCAR3, or COV362 cells (*n =* 6). *P < 0.05; **P < 0.01; ***P < 0.005; ****P < 0.0001, by log-rank test for survival (**F**) and unpaired, 2-tailed Student’s *t* test for the other panels.

To identify novel drivers of stemness in OC, we performed ATAC-seq in flow-sorted CSCs (ALDH^+^CD133^+^) versus non-CSCs (ALDH^–^CD133^–^) derived from OVCAR5 cells. The TF-encoding *FOXK2* gene was found among the top genes associated with open chromatin peaks (Supplemental Figure 1A; supplemental material available online with this article; https://doi.org/10.1172/JCI151591DS1). The expression level of *FOXK2* was indeed upregulated in flow-sorted ALDH^+^ versus ALDH^–^ cells from primary high-grade serous OC (HGSOC) specimens (*n =* 5, *P =* 0.008) and from the cell lines OVCAR5 (*n =* 3, *P =* 0.03) and COV362 (*n =* 3, *P =* 0.02) (Figure 1, A and B), and in ALDH^+^ cell–enriched tumor cell spheroids compared with monolayer cultures (Figure 1C) derived from OVCAR5 and COV362 cell lines.

To assess the broader significance of FOXK2 to OC, its expression level was measured in OC cell lines relative to FT190 (normal fallopian tube epithelial [FTE]) cells and normal endometrial (NoEM) cells. FOXK2 was upregulated in all OC cell lines compared with FT190 and NoEM cells at both mRNA and protein levels (Supplemental Figure 1B). FOXK2 expression was also measured by immunohistochemistry (IHC) in a set of residual HGSOC tumors collected after 3 to 6 cycles of neoadjuvant chemotherapy (NACT), which are enriched in CSCs (22). We observed increased expression of FOXK2 (measured as H-score) in post-NACT tumors (*n =* 19) when compared with FTE (control, *n =* 6) (Figure 1D and Supplemental Figure 1C, *P =* 0.003). Further, exploration of The Cancer Genome Atlas (TCGA) and Genotype-Tissue Expression (GTEx) databases revealed higher *FOXK2* expression in human OC specimens (OV, *n =* 427) compared with normal FTE (*n =* 5; Figure 1E). *FOXK2* expression levels correlated with clinical outcomes, as shown by longer progression-free survival (PFS) in patients whose tumors exhibited low *FOXK2* expression (bottom 25th percentile, *n =* 155, median PFS = 20 months) compared with those with high *FOXK2* expression (top 25th percentile, *n =* 154, median PFS = 14 months; *P =* 0.015) (Figure 1F). Altogether, the data support the idea that FOXK2 expression is increased in OC cells and tumors and is highly transcribed in CSCs.

### FOXK2 regulates stemness in OC cells.

To investigate the functions of FOXK2 in CSCs, we generated a knockdown model by stably transducing lentiviral particles containing *FOXK2*-targeting short hairpin RNA (shRNA) sequences (shFOXK2) in OVCAR5, OVCAR3, and COV362 cell lines and in primary HGSOC cells. FOXK2 expression was reduced at mRNA and protein levels in cells transduced with shFOXK2 versus control shRNA (Figure 1G and Supplemental Figure 1D). FOXK2 knockdown led to decreased ALDH^+^ cell populations in OVCAR5 (*n =* 3, *P =* 0.03 in both shFOXK2-1 and shFOXK2-2), OVCAR3 (*n =* 3, *P =* 0.0003 in shFOXK2-1 and 0.0005 in shFOXK2-2), and COV362 (*n =* 3, *P =* 0.03 in shFOXK2-1 and 0.0045 in shFOXK2-2), as assessed by flow cytometry analysis (Figure 1H). Further, sphere-forming ability was significantly reduced in OVCAR5 (*n =* 6, *P =* 0.001 in shFOXK2-1 and *P* < 0.0001 in shFOXK2-2), OVCAR3 (*n =* 6, *P <* 0.0001 in shFOXK2-1 and *P* < 0.0001 in shFOXK2-2) and COV362 (*n =* 6, *P =* 0.002 in shFOXK2-1 and *P* < 0.0001 in shFOXK2-2) cells stably transduced with shFOXK2 versus control shRNA (Figure 1I and Supplemental Figure 1E). We also transduced shFOXK2 directly into ALDH^+^ cells flow sorted from OVCAR5 cells and confirmed the knockdown (Supplemental Figure 1F). ALDH^+^ cells transduced with shFOXK2 displayed significantly reduced sphere-forming ability (Supplemental Figure 1G), suggesting inhibition of stemness features.

**Figure 2:**
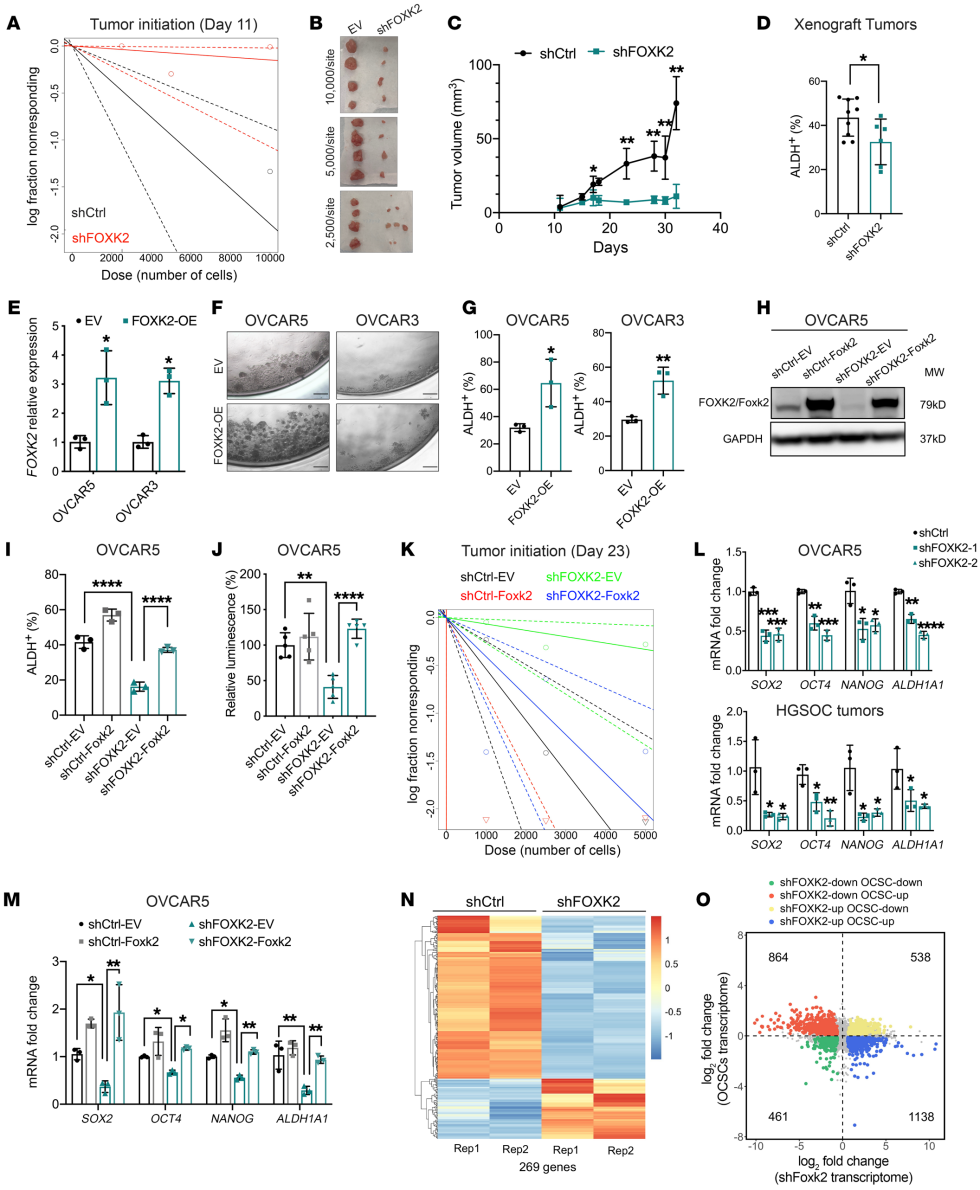
FOXK2 regulates tumor initiation and stemness gene expression in OC cells. (**A**) Log-fraction plot of serial dilutions of shCtrl and shFOXK2 OVCAR5 cells (*n =* 4 mice/group) estimated by ELDA. (**B**) Xenografts collected from mice in indicated groups (*n =* 4 mice/group). (**C**) Growth curves of xenografts from the 5000 cells/mice group in **B** (*n =* 4 mice). (**D**) Percentage of ALDH^+^ cells determined by flow cytometry in xenografts generated by shCtrl (*n =* 8) and shFOXK2 (*n =* 6) cells. (**E** and **F**) *FOXK2* mRNA levels (*n =* 3) (**E**) and representative images of spheroids (original magnification, ×20) (*n =* 6) (**F**) of OVCAR5 and OVCAR3 cells transfected with empty vector (EV) or FOXK2 expression vector (FOXK2-OE). (**G**) Percentages of ALDH^+^ cells in EV- or FOXK2-OE–transduced OC cells (*n =* 3). (**H**) Western blot of protein levels of FOXK2/Foxk2 in OVCAR5 shCtrl and shFOXK2 cells transduced with EV (shCtrl-EV, shFOXK2-EV) or Foxk2 (shCtrl-Foxk2, shFOXK2-Foxk2) (*n =* 3). (**I** and **J**) Percentages of ALDH^+^ CSCs (*n =* 3) (**I**) and cell viability in spheroid cultures (*n =* 6) formed from OVCAR5 shCtrl and shFOXK2 cells transduced with EV or Foxk2. (**K**) Log-fraction plot of serial dilutions of shCtrl and shFOXK2 cells transduced with EV or Foxk2 (*n =* 4 mice/group) generated from ELDA. (**L** and **M**) mRNA levels of *SOX2*, *OCT4*, *NANOG*, and *ALDH1A1* in shCtrl- or shFOXK2-transduced OVCAR5 cells (*n =* 3) and cells from HGSOC tumors (*n =* 3) (**L**), and in OVCAR5 shCtrl and shFOXK2 cells transduced with EV or Foxk2 (*n =* 3) (**M**). (**N**) Heatmap shows differentially expressed genes (DEGs) measured by RNA-seq in shCtrl versus shFOXK2 cells (269 genes, *n =* 2). (**O**) Scatter plot shows overlapping genes among DEGs in CSCs (ALDH^+^CD133^+^) vs non-stem cells (ALDH^–^CD133^–^) (*n =* 2) and shCtrl versus shFOXK2 OVCAR5 cells (*n =* 2). *P < 0.05; **P < 0.01; ***P < 0.005; ****P < 0.0001, by unpaired, 2-tailed Student’s *t* test when comparing 2 groups and 2-way ANOVA with Tukey’s multiple comparisons test when comparing more than 2 groups.

To assess the effects of FOXK2 on TIC in vivo, we performed a limiting-dilution experiment by injecting serially diluted numbers of OVCAR5 cells (10,000, 5000, and 2500) transduced with shFOXK2 versus control shRNA in nude mice. The extreme limiting-dilution analysis (ELDA) calculations indicated that the shFOXK2 group contained significantly fewer CSCs compared with the control group (shFOXK2 1:67,469 vs. shCtrl 1:5281, *P =* 0.002; Figure 2A and Supplemental Table 1). Tumor initiation was delayed in the group transduced with shFOXK2 compared with control (8 of 12 in shCtrl group vs. 1 of 12 in shFOXK2 on day 11, *P* = 0.0032; Supplemental Table 2). Tumors derived from shFOXK2-transduced OVCAR5 cells displayed slower tumor growth (Figure 2C; *P =* 0.003 on day 23; *P =* 0.001 on day 32) and smaller tumor weights at the endpoint compared with control xenografts (Figure 2B and Supplemental Figure 2A; *P <* 0.0001). Knockdown efficiency was confirmed in xenografts by quantitative real-time PCR (qRT-PCR) (Supplemental Figure 2B) and IHC (Supplemental Figure 2C). Percentages of ALDH^+^ cells and sphere-forming capability of cells dissociated from xenografts were decreased in shFOXK2 versus control tumors (Figure 2D; *P =* 0.04; Supplemental Figure 2, D and E).

Further, we generated OC and FTE cells stably overexpressing FOXK2 (FOXK2-OE). FOXK2 overexpression was confirmed at mRNA and protein levels compared to cells transfected with empty vector (EV) (Figure 2E and Supplemental Figure 2F). FOXK2-OE cells formed spheroids greater in size and number compared with EV-transduced cells (Figure 2F and Supplemental Figure 2G; *n =* 6; OVCAR5 *P <* 0.0001; OVCAR3 *P <* 0.0001). Percentages of ALDH^+^ cells were increased in FOXK2-OE compared with EV-transduced cells (Figure 2G and Supplemental Figure 2H; OVCAR5 *P =* 0.03; OVCAR3 *P =* 0.008). Likewise, FOXK2 was stably transduced in immortalized FTE cells (FT190) and in NoEM cells, which harbor low FOXK2 expression levels (Supplemental Figure 2I). Spheroid-forming assays demonstrated that overexpression of FOXK2 promoted the growth of spheroids (Supplemental Figure 2, J and K), consistent with the phenotype observed in OC cell lines.

To exclude the off-target effects of FOXK2 knockdown, we restored the function of FOXK2 by overexpressing murine Foxk2, which has 95% similarity with the human protein, but is not targeted by shFOXK2 (Supplemental Figure 3A). Foxk2 mRNA and protein expression levels were increased in shFOXK2-Foxk2 cells compared with EV-transduced cells (Figure 2H and Supplemental Figure 3, B–D). The ALDH^+^ population was restored in shFOXK2-Foxk2 cells compared with shFOXK2-EV cells (Figure 2I and Supplemental Figure 3E). Likewise, FOXK2-knockdown cells transduced with Foxk2 had increased spheroid-forming capacity compared with EV-transduced cells (Figure 2J and Supplemental Figure 3, F and G) and increased TIC, as measured by injection of serial dilutions of engineered cells (Figure 2K and Supplemental Table 3). ELDA-based calculations support the notion that shFOXK2-Foxk2 cells contain higher numbers of CSCs compared with the shFOXK2-EV cells (shFOXK2-Foxk2 1:2449 vs. shFOXK2-EV 1:15,039, *P =* 0.0105; Supplemental Table 4). CSC frequency was also significantly different between shCtrl-Foxk2 and shCtrl-EV groups (*P =* 0.0091) and in shCtrl-EV versus shFOXK2-EV groups (*P =* 0.0038; Supplemental Table 4). TIC was restored by transduction of *Foxk2* compared with control (8 of 12 in shFOXK2-Foxk2 group vs. 2 of 12 in shFOXK2-EV on day 23, *P =* 0.013; Supplemental Table 3). Flow cytometry analysis of cells dissociated from tumors harvested from this experiment indicated that the ALDH^+^ CSC population was restored in the shFOXK2-Foxk2 group compared with the shFOXK2-EV group (Supplemental Figure 3H). Taken together, the results support the idea that the observed reduction in ovarian CSCs was induced by FOXK2 knockdown.

Additionally, FOXK2 knockdown significantly reduced mRNA expression levels of stemness-associated TFs (*SOX2*, *OCT4*, and *NANOG*) and stemness marker *ALDH1A1* in OC cell lines and primary cells dissociated from a human ovarian tumor (Figure 2L and Supplemental Figure 4A). Xenograft tumors from OVCAR5 shFOXK2 also had decreased expression levels of stemness-associated TFs and *ALDH1A1* (Supplemental Figure 4B), consistent with observations derived from OC cell lines. Rescuing the FOXK2 function by overexpressing *Foxk2* restored the expression of stemness-associated genes in shFOXK2 cells (Figure 2M and Supplemental Figure 4C). On the other hand, expression of stemness-associated TFs was significantly increased in FOXK2-OE OC cells (OVCAR5 and OVCAR3) and noncancer cells (FT190 and NoEM) compared with cells transfected with EV (Supplemental Figure 4, D and E). Together, these data strongly support the hypothesis that FOXK2 regulates cancer stemness.

To assess the broader effects of FOXK2 on the transcriptome, we performed RNA sequencing (RNA-seq) in OVCAR5 cells stably transduced with shFOXK2 and control shRNA. A total of 7410 genes were differentially expressed between OVCAR5 stably transduced with shFOXK2 versus control shRNA (FDR < 0.05), of which 3533 genes were downregulated and 3877 were upregulated (Supplemental Tables 5 and 6). Gene set enrichment analysis (GSEA) identified significant enrichment in the “adult tissue stem module” in shCtrl cells compared with shFOXK2 cells, a gene set related to an embryonic stem cell–like transcriptional program bearing similar characteristics to those of CSCs (ref. 23 and Supplemental Figure 4F). A heatmap shows a clear distinction between shCtrl and shFOXK2 cells for the expression levels of 269 genes included in the “adult tissue stem module” (Figure 2N). Enrichment in “cultured stem cells” and “epithelial-mesenchymal transition” gene sets was also reduced in OC cells transduced with shRNA targeting FOXK2 (Supplemental Figure 4F). When integrated with the results of RNA-seq analysis comparing OVCAR5 ALDH^+^CD133^+^ (CSCs) vs. ALDH^–^CD133^–^ (non-CSCs), 3001 differentially expressed genes (DEGs) overlapped. Among them, 864 genes upregulated in CSCs were downregulated in cells transduced with shRNA targeting FOXK2, while 1138 genes downregulated in CSCs were upregulated in shFOXK2 cells (Figure 2O). Together, 2002 of 3001 DEGs in shFOXK2 cells displayed opposite differential expression compared with CSCs, indicating that knockdown of this TF significantly impacts the stemness-associated transcriptome. Further, GSEA of RNA-seq results of 427 OC tumor samples in TCGA identified enrichment of “KEGG pathways in cancer,” “breast cancer progenitors,” and “embryonic stem cells early stage” gene sets in specimens with high FOXK2 expression versus those with low FOXK2 expression, further supporting an association between FOXK2 and a stemness phenotype (Supplemental Figure 4G).

### FOXK2 directly regulates the ERN1 gene.

**Figure 3:**
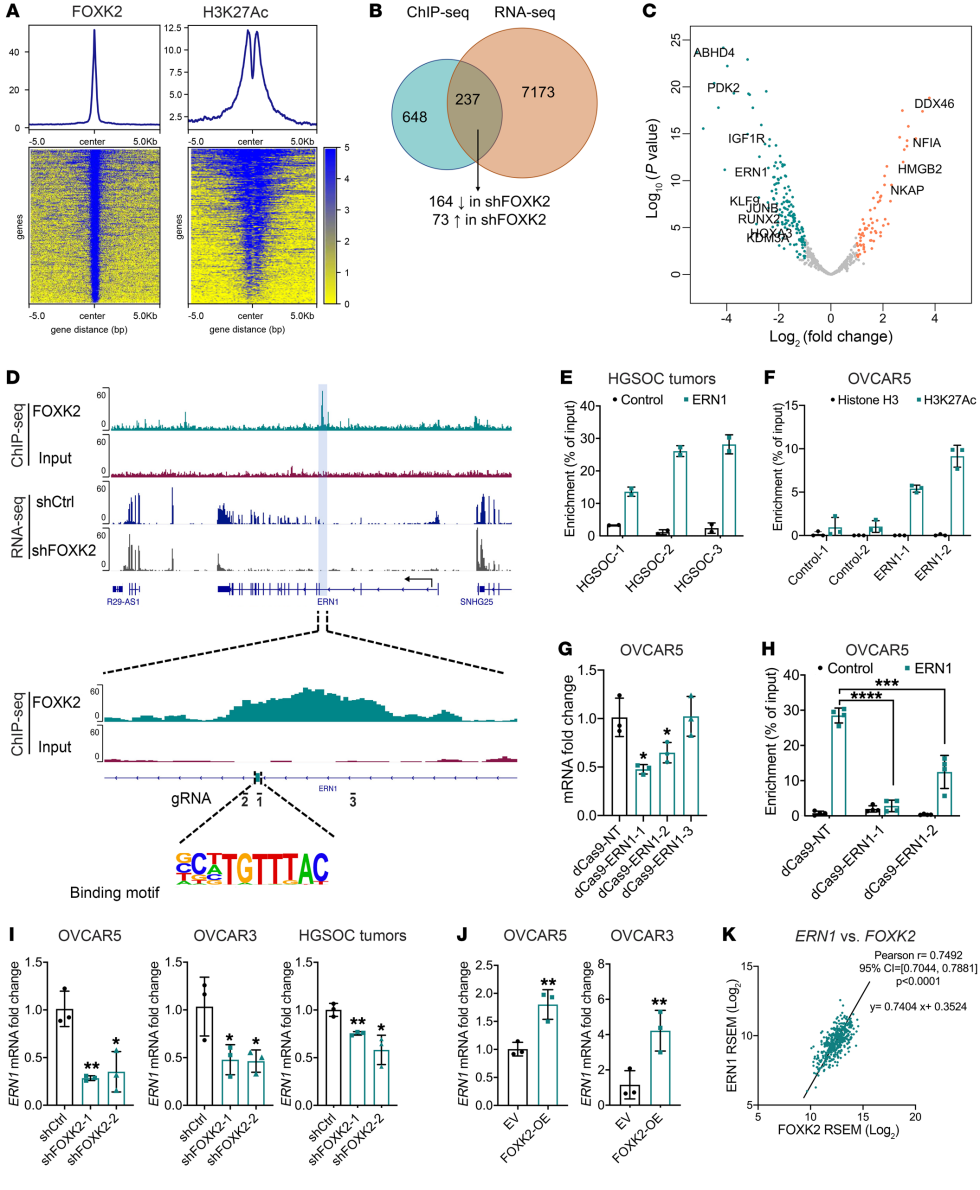
FOXK2 directly regulates IRE1α expression in OC cells. (**A**) Density plots (upper) and heatmaps (lower) of normalized FOXK2 and H3K27Ac ChIP-seq reads at regions differentially bound by FOXK2 in OVCAR5. (**B**) Venn diagram shows numbers of overlapping genes FOXK2 peaks in ChIP-seq (FDR < 0.05) and DEGs in RNA-seq (log_2_[fold change] > 2, FDR < 0.05) in OVCAR5 transduced with shFOXK2-2 (shFOXK2) versus shCtrl. (**C**) Volcano plot of overlapping genes described in **B**. (**D**) Integrative Genomics Viewer (IGV, https://software.broadinstitute.org/software/igv/) tracks of the FOXK2 binding peak in the *ERN1* gene, and *ERN1* mRNA by RNA-seq in shCtrl and shFOXK2 cells. The FOXK2 binding motif is indicated along with the position of gRNA sequences used (1, 2, 3). (**E**) ChIP-qPCR shows binding of FOXK2 to the *ERN1* gene in HGSOC tumors (*n =* 3). Amplification of a sequence 1 kb downstream was used as a control. (**F**) ChIP-qPCR measured enrichment of H3K27Ac in the FOXK2 binding site of the *ERN1* gene (*n =* 3). Amplification of a sequence 1 kb downstream was used as a control. (**G**) *ERN1* mRNA levels in OVCAR5 transduced with nontargeting dCas9-gRNA (dCas9-NT) or dCas9-sgRNA targeting the FOXK2 binding motif on *ERN1* (dCas9-ERN1-1 through -3) (*n =* 3). The position of target sequences for gRNAs is indicated in **D**. (**H**) ChIP-qPCR using the same primers and control as in **E** shows binding of FOXK2 to the *ERN1* gene in OVCAR5 transduced with dCas9-NT, dCas9-ERN1-1, or dCas9-ERN1-2. (**I** and **J**) *ERN1* mRNA levels in shCtrl- and shFOXK2-transduced OC cells and HGSOC tumors (*n =* 3) (**I**) and in EV- or FOXK2-OE–transfected OC cells (*n =* 3) (**J**). (**K**) Scatter plot shows the correlation between mRNA levels of *FOXK2* and *ERN1* in ovarian tumors profiled by TCGA (*n =* 427). *P < 0.05; **P < 0.01; ***P < 0.005; ****P < 0.0001, by unpaired, 2-tailed Student’s *t* test.

To identify targets of FOXK2, we performed ChIP-seq using an antibody directed against FOXK2 and incorporated with ChIP-seq of acetylated histone H3 on lysine 27 (H3K27Ac) in OVCAR5 from published data (24). The density plot at the identified FOXK2 peaks shows strong ChIP-seq signal compared with background, indicating the specificity of FOXK2 ChIP (Figure 3A). The density of the H3K27Ac modification mark at the FOXK2 binding sites showed strong H3K27Ac signals around FOXK2 peaks. Furthermore, we observed a dip in the H3K27Ac signal around the FOXK2 binding sites, indicating displaced nucleosomes in these regions and supporting the notion that FOXK2 created a nucleosome-free region at the binding sites (Figure 3A). Motif analysis verified that peaks were enriched with the known FOXK2 binding motif, TGTTTAC (Supplemental Figure 5A). The majority of FOXK2’s binding peaks were in promoter, intron, and intergenic regions (Supplemental Figure 5B). By integrating ChIP-seq peaks with the DEGs between OVCAR5 cells transduced with shRNA targeting FOXK2 versus control, we identified 237 genes that were both differentially expressed and contained FOXK2 binding sites, suggesting these genes could be FOXK2 targets. Of those, 164 genes were downregulated in shFOXK2 cells (Figure 3B and Supplemental Table 7), representing potential direct targets. Among them, we detected several known FOXK2 targets, such as *KDM3A*, *ATXN1*, *IGF1R*, and *KLF9* (Figure 3C and ref. 25).

Among the potential direct targets, *ERN1* was one of the top genes. Peak analysis showed the FOXK2 binding motif in intron 2 of the *ERN1* gene at +47,839 bp from the transcription start site (TSS) and decreased *ERN1* expression in shFOXK2-transduced cells compared with controls, according to RNA-seq analysis (Figure 3D and Supplemental Table 8). To further verify the binding of FOXK2 to *ERN1* in primary OC specimens, we performed ChIP-qPCR. FOXK2 binding to the same region of the *ERN1* gene was confirmed by qPCR with primers flanking intron 2 of *ERN1* (chr17: 64,082,103–64,082,506) (Figure 3E). Further, FOXK2 binding peaks in the same *ERN1* region were confirmed in the ENCODE data sets previously recorded for other cell lines (K362, HepG2, GM12878, and HEK293T; Supplemental Figure 5C and refs. 26, 27). The peaks indicated binding of FOXK2 to the same region of the *ERN1* gene in cells of different tissue origin, suggesting conserved regulation of the *ERN1* gene by FOXK2. FOXK2 peaks overlapped with active histone mark H3K27Ac on the *ERN1* gene, as mapped in the OVCAR5 cells from previously published results (24) and in overlaid H3K27Ac ChIP-seq profiles of 7 cell lines from the ENCODE project (ref. 26 and Supplemental Figure 5C), suggesting that this region is a potential enhancer. ChIP-qPCR using an antibody against H3K27Ac and primers flanking the same region confirmed enrichment of this active histone mark in the same region of *ERN1* where FOXK2 was also immunoprecipitated, supporting the hypothesis that this binding site distal to the TSS is a regulatory region (Figure 3F).

To characterize the regulatory role of FOXK2 binding to this *ERN1* enhancer, we used an endonuclease-deficient Cas9 (dCas9) system to disrupt FOXK2 binding to this site. To this end, we designed 4 distinct sgRNAs: 2 of them targeting the FOXK2 motif (dCas9-ERN1-1 and dCas9-ERN1-2), 1 targeting a sequence 250 bp downstream of the motif (dCas9-ERN1-3), and 1 non-genome-targeting control sgRNA (dCas9-NT) (Figure 3D). Notably, we observed a significant reduction in *ERN1* mRNA with dCas9-ERN1-1 and dCas9-ERN1-2. On the other hand, dCas9-ERN1-3 and dCas9-NT did not yield any detectable alterations in *ERN1* mRNA (Figure 3G). These findings indicate that targeting the FOXK2 motif with dCas9 reduces *ERN1* expression by potentially blocking FOXK2 binding. To further verify this, we performed ChIP-qPCR to measure FOXK2 enrichment before and after perturbations with dCas9. Notably, we detected significant reduction in FOXK2 enrichment in cells transduced with dCas9-ERN1-1 and dCas9-ERN1-2 (Figure 3H), demonstrating that FOXK2 binding to the distal regulatory element is critical for *ERN1* expression.

To further validate that *ERN1* is directly regulated by FOXK2 in different settings, we examined its expression in additional cell lines in which FOXK2 was either knocked down or overexpressed. *ERN1* was downregulated in shFOXK2-transduced OVCAR5 and OVCAR3 cells and human HGSOC tumor cells (Figure 3I) compared with control cells and tumors. *ERN1* expression was also significantly increased in FOXK2-OE OC (OVCAR5 and OVCAR3) and non-cancer (FT190 and NoEM) cells (Figure 3J and Supplemental Figure 6C). Furthermore, expression levels of *FOXK2* and *ERN1* were significantly correlated with each other in HGSOC tumors profiled in TCGA data set (Pearson’s *r* = 0.7492, *P <* 0.0001, *n =* 427; Figure 3K). Together, these data establish that *ERN1* is a direct FOXK2 target.

### FOXK2 regulates the expression of IRE1α involved in the UPR.

**Figure 4:**
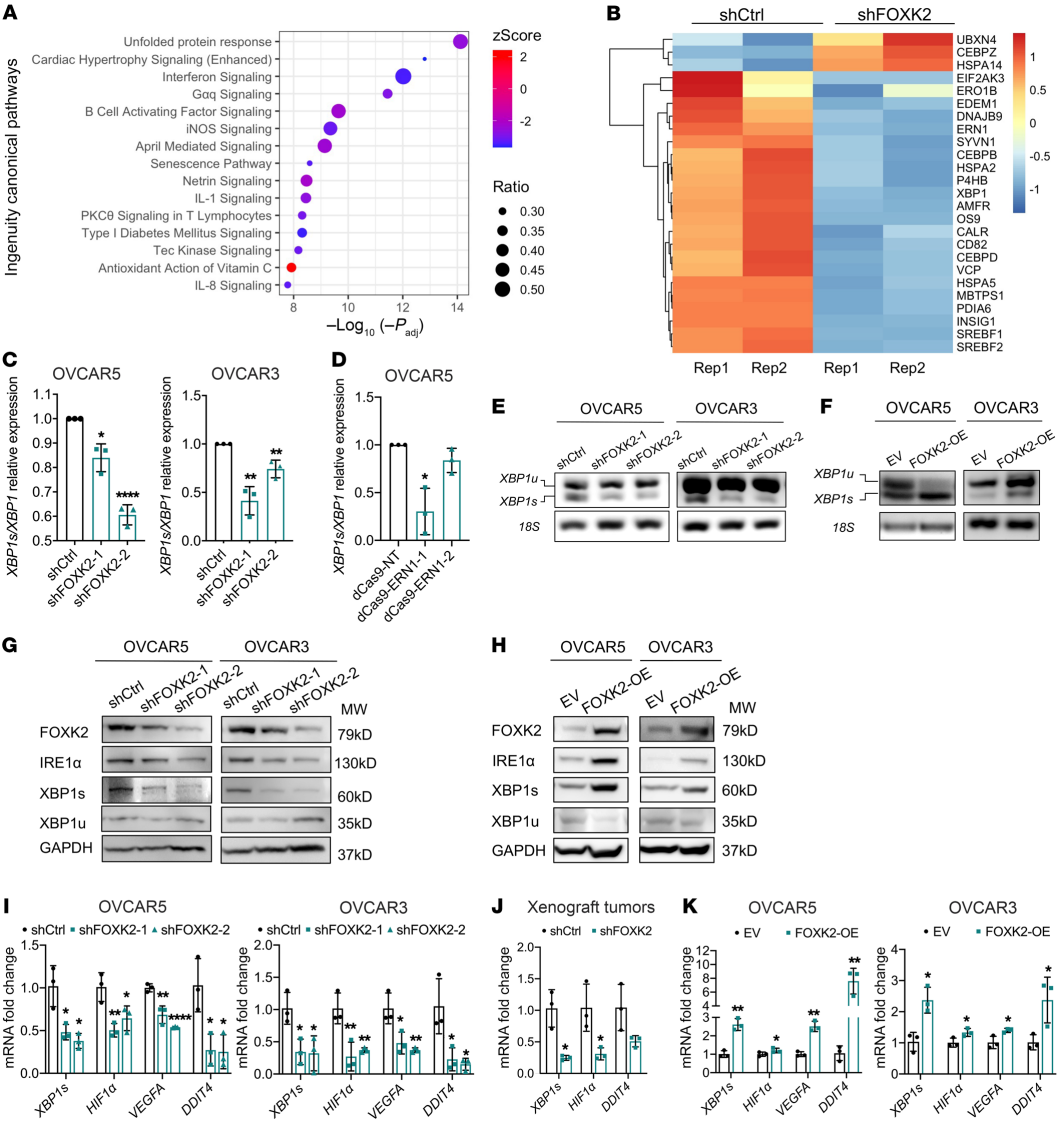
FOXK2 directly regulates IRE1α and activates the unfolded protein response. (**A**) Top 15 canonical pathways identified by Ingenuity Pathway Analysis (IPA) among DEGs determined by RNA-seq in OVCAR5 transduced with shFOXK2-2 (shFOXK2) or control shRNAs (shCtrl). (**B**) Heatmap shows mRNA expression levels (RNA-seq) of 25 genes involved in the UPR pathway in shCtrl and shFOXK2 OVCAR5 cells. (**C** and **D**) Ratios measured by qRT-PCR (*n =* 3) of the *XBP1* mRNA spliced isoform (*XBP1s*) relative to the unspliced *XBP1* (*XBP1u*) in shCtrl- and shFOXK2-transduced OVCAR5 and OVCAR3 cells (**C**), and in OVCAR5 cells transduced with dCas9-NT or dCas9-ERN1-1/2 (**D**). (**E** and **F**) RT-PCR products resolved by agarose gel electrophoresis of the *XBP1u* and the *XBP1s* in shCtrl- and shFOXK2-transduced OVCAR5 and OVCAR3 cells (**E**) and in EV (control) and FOXK2-overexpressing (FOXK2-OE) OVCAR5 and OVCAR3 cells (**F**). (**G** and **H**) Western blot of FOXK2, IRE1α, spliced XBP1 (XBP1s), unspliced XBP1 (XBP1u), and GAPDH in shCtrl- and shFOXK2-transduced OC cells (*n =* 3) (**G**), and in OVCAR3 and OVCAR5 cells transduced with EV or FOXK2-OE (*n =* 3) (**H**). (**I**) qRT-PCR–measured mRNA levels (*n =* 3) of *XBP1s*, *HIF1α*, *VEGFA*, and *DDIT4* in shCtrl- and shFOXK2-transduced OVCAR5 and OVCAR3 cells. (**J**) mRNA levels (*n =* 3) of *XBP1*, *HIF1α*, and *DDIT4* measured by qRT-PCR in xenografts derived from shCtrl or shFOXK2 OVCAR5 cells. (**K**) mRNA expression levels (*n =* 3) of *XBP1s*, *HIF1α*, *VEGFA*, and *DDIT4* in OVCAR5 and OVCAR3 transfected with EV or FOXK2-OE. *P < 0.05; **P < 0.01; ****P < 0.0001, by unpaired, 2-tailed Student’s *t* test.

At the transcriptomic level, the top pathway downregulated in OVCAR5 cells transduced with shFOXK2 was the UPR pathway (Figure 4A). A heatmap containing genes in the UPR pathway displays downregulation of transcripts related to the UPR, including *ERN1* and IRE1α’s substrate *XBP1* in shFOXK2-transduced OC cells (Figure 4B). Aside from *ERN1*, FOXK2 was found to bind to regulatory regions of other genes associated with UPR and ER homeostasis, such as *DDIT4* and *JUNB* (refs. 20, 28, and Supplemental Figure 5D).

The major function of *ERN1*’s protein product, IRE1α, is to catalyze the mRNA-splicing reaction of *XBP1*, ultimately yielding the spliced form XBP1s, an active TF, which regulates the transcription of several key genes involved in the UPR (29). To confirm the effects of FOXK2 on the UPR, we measured the ratio of spliced (*XBP1s*) versus total *XBP1* mRNA levels in OC cells in which FOXK2 was either knocked down or overexpressed. The ratio was decreased in shFOXK2-transduced OVCAR5 and OVCAR3 cells compared with controls (Figure 4C). In OVCAR5 cells transduced with gRNA targeting FOXK2 binding sites on *ERN1*, the ratio of *XBP1s* to total *XBP1* mRNA was also reduced compared with cells transduced with nontargeting gRNA (dCas9-NT) (Figure 4D). The splicing assay also indicated that the expression levels of *XBP1s* mRNA were reduced in shFOXK2-transduced OVCAR5 and OVCAR3 cells compared with controls and augmented in FOXK2-OE cells versus EV-transduced cells (Figure 4, E and F, and Supplemental Figure 7A). Western blotting confirmed downregulation of IRE1α and XBP1s at the protein level in OC cells transduced with shFOXK2 versus control shRNA (Figure 4G) and upregulation in FOXK2-OE versus control cells (Figure 4H). Further, the expression of known XBP1 target genes (20)*, HIF1α*, *VEGFA*, and *DDIT4*, was decreased significantly in OVCAR5 and OVCAR3 cells stably transduced with shFOXK2 and in xenografts derived from OVCAR5-shFOXK2 cells compared with controls (Figure 4, I and J). On the other hand, these target genes were upregulated in FOXK2-OE versus EV-transduced OC or non-cancer cells (Figure 4K and Supplemental Figure 6D). Together, these results establish the role of FOXK2 in regulating the UPR pathway by directly altering IRE1α/XBP1s levels.

As the UPR pathway includes 3 major branches, IRE1α/XBP1, PERK/eIF2α/ATF4, and ATF6 (30), we also examined whether the other 2 branches were affected by the level of FOXK2 expression. PERK/eIF2α/ATF4 and the ATF6 branch were not altered by FOXK2 knockdown in OVCAR5 and OVCAR3 (Supplemental Figure 6, A and B). However, the expression of GRP78, a key upstream of regulator of the UPR, was decreased in shFOXK2 compared with shCtrl-transduced OVCAR5 and OVCAR3 cells (Supplemental Figure 6, A and B), possibly due to a feedback regulatory effect of IRE1α on GRP78, as previously reported (31, 32).

### IRE1α/XBP1s is associated with stemness in OC.

**Figure 5:**
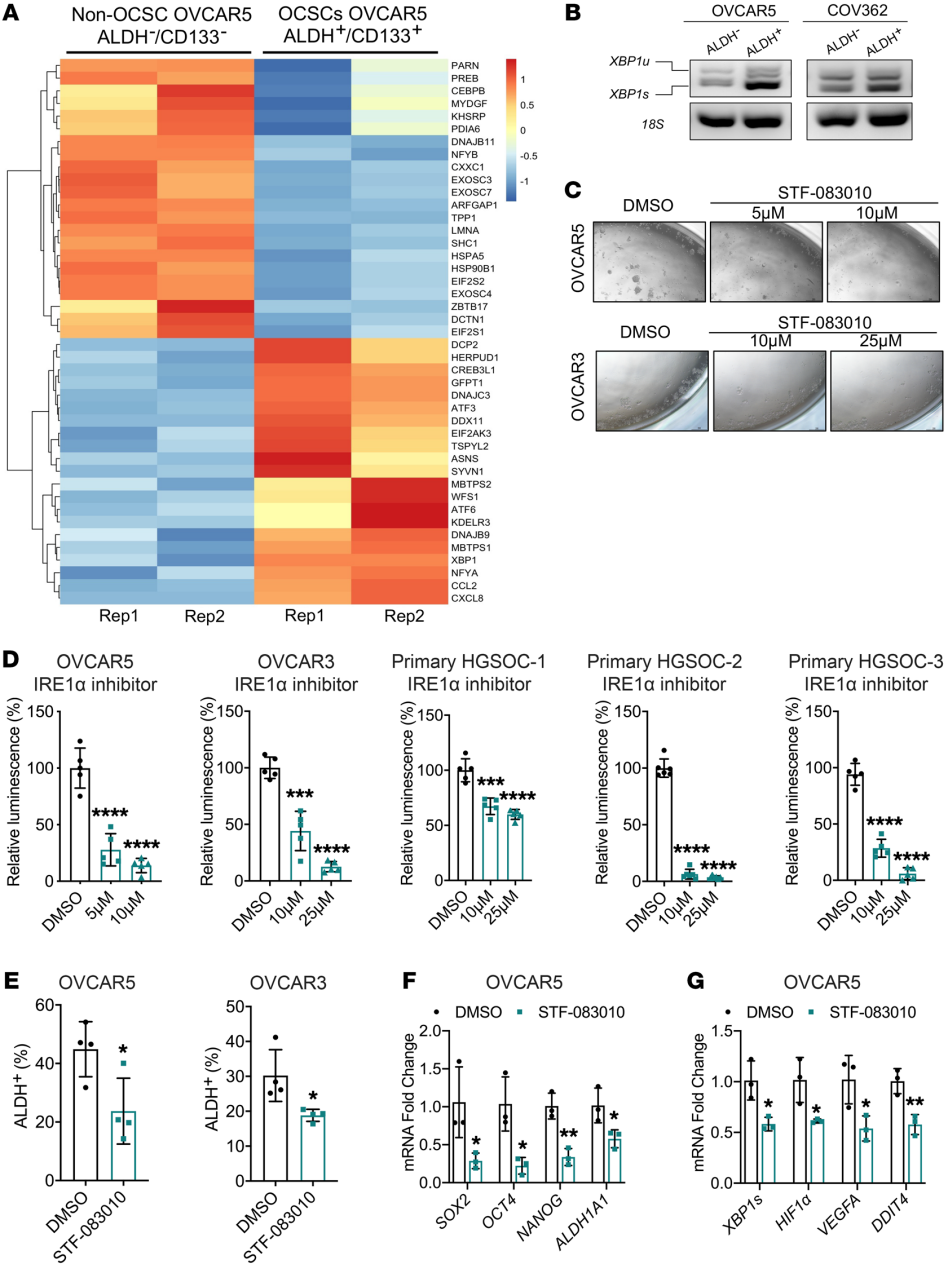
IRE1α/XBP1s promotes stemness features. (**A**) Heatmap shows levels of DEGs (from RNA-seq) among those listed in the “hallmark of unfolded protein response” GSEA gene set in ovarian CSCs (ALDH^+^CD133^+^) versus non-CSCs (ALDH^–^CD133^–^) sorted by FACS from OVCAR5 cells (*n =* 2). (**B**) RT-PCR products resolved by agarose gel electrophoresis of the full-length *XBP1* transcript (*XBP1u*) and the spliced isoform (*XBP1s*) in ALDH^+^ and ALDH^–^ cells sorted by FACS from OVCAR5 and COV362 cells. (**C**) Representative pictures of spheroids formed from OVCAR5 and OVCAR3 cells treated with the IRE1α inhibitor STF-083010 (STF) or DMSO (original magnification, ×20) (*n =* 5). (**D**) Effects of IRE1α inhibition on spheroid formation assessed by measuring cell viability in OVCAR5 and OVCAR3 cells (*n =* 5 cultures) and in cells isolated from 3 HGSOC tumors (*n =* 5 per dose). (**E**) Percentage of ALDH^+^ cells measured by flow cytometry (*n =* 3) in OVCAR5 and OVCAR3 cells treated with STF-083010 or vehicle (DMSO). (**F**) mRNA expression levels of *SOX2*, *OCT4*, *NANOG*, and *ALDH1A1* measured by qRT-PCR in OVCAR5 cells treated with STF-083010 or DMSO (*n =* 3). (**G**) qRT-PCR–measured mRNA expression levels (*n =* 3) of *XBP1s*, *HIF1α*, *VEGFA*, and *DDIT4* in OVCAR5 cells treated with STF-083010 or DMSO. *P < 0.05; **P < 0.01; ***P < 0.005; ****P < 0.0001, by unpaired, 2-tailed Student’s *t* test.

To further interrogate the function of IRE1α relative to stemness, we analyzed the expression of UPR-associated genes among the DEGs between CSCs and non-CSCs, noting clear separation, as illustrated by the heatmap in Figure 5A. Genes in the UPR pathway were distinctly expressed in CSCs versus non-CSCs, including multiple critical downstream genes, such as *XBP1*, *ATF3*, *ATF6*, *PDK1*, *HIF1α*, and *VEGFA* (Supplemental Figure 7B). Splicing of *XBP1* mRNA was measured in flow-sorted ALDH^+^ versus ALDH^–^ cells, and increased *XBP1* splicing was noted in ALDH^+^ compared with ALDH^–^ cells (Figure 5B). GSEA of TCGA OC samples (*n =* 427) also showed enrichment of UPR genes in *FOXK2*-high versus *FOXK2*-low tumor specimens (Supplemental Figure 7C). Further, spheroid cultures derived from OVCAR5, OVCAR3, and COV362 cells, which are enriched in ALDH^+^ cells, harbored higher expression of *ERN1* compared with monolayer cultures (Supplemental Figure 7D), as well as a higher *XBP1s*/*XBP1* ratio (Supplemental Figure 7E). Additionally, expression of other IRE1α/XBP1s-target genes, including *XBP1s*, *HIF1α*, *DDIT4*, and *JMJD1A*, was increased in ALDH^+^ cell–enriched tumor spheroids (Supplemental Figure 7F), suggesting that the UPR pathway was activated in spheroid cultures and in CSCs.

**Figure 6:**
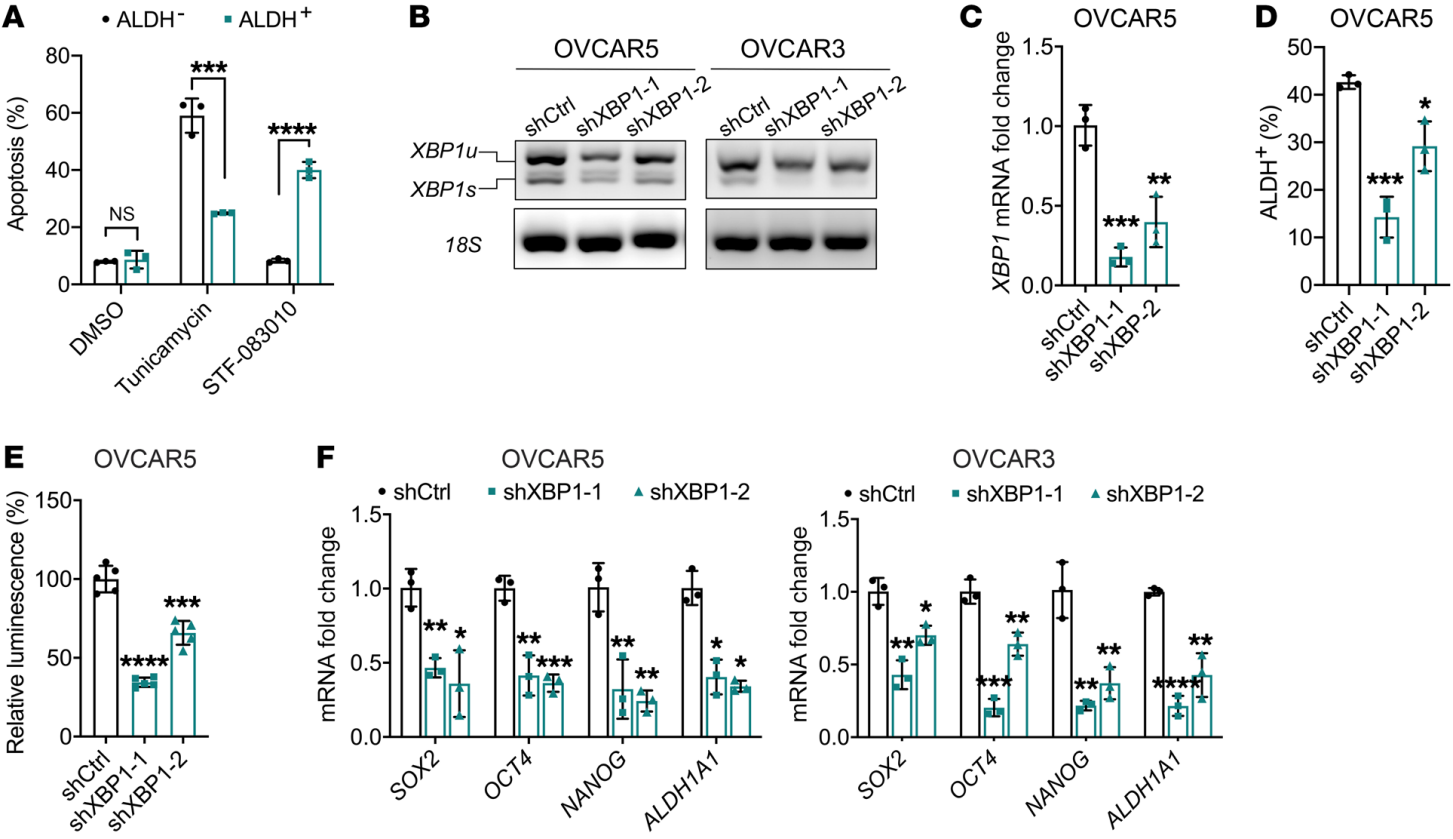
XBP1 splicing is associated with stemness characteristics. (**A**) Annexin V staining and flow cytometry–measured apoptotic cells among sorted ALDH^+^ and ALDH^–^ OVCAR5 cells treated with DMSO, 2 μg/mL tunicamycin, or 10 μM STF-083010 (*n =* 3). (**B**) qRT-PCR determination of the full-length *XBP1* transcript (*XBP1u*) and the spliced isoform (*XBP1s*) in OVCAR5 and OVCAR3 cells transduced with 2 different shRNAs directed at *XBP1* (shXBP1) or with control shRNA (shCtrl). (**C**) *XBP1* mRNA expression levels measured by qRT-PCR (*n =* 3) in shCtrl and shXBP1 OVCAR5 cells. (**D**) Percentage of ALDH^+^ cells (*n =* 3) in shXBP1- and shCtrl-transduced OVCAR5 cells. (**E**) Spheroid formation assessed by a cell viability assay in shCtrl- and shXBP1-transduced OVCAR5 cells (*n =* 5). (**F**) mRNA expression levels (*n =* 3) of *SOX2*, *OCT4*, *NANOG*, and *ALDH1A1* in shCtrl- and shXBP1-transduced OVCAR5 and OVCAR3 cells measured by qRT-PCR. *P < 0.05; **P < 0.01; ***P < 0.005; ****P < 0.0001, by unpaired, 2-tailed Student’s *t* test.

Our observations indicating activated UPR signaling, regulated by IRE1α/XBP1 in ALDH^+^ CSCs, suggest that this mechanism may be necessary to protect ALDH^+^ cells from ER stress in the tumor microenvironment. FACS-isolated ALDH^+^ and ALDH^–^ cells were treated with tunicamycin to induce ER stress and the ensuing apoptosis was assessed. Increased numbers of ALDH^+^ ovarian CSCs survived after induction of ER stress by tunicamycin compared with ALDH^–^ cells (Figure 6A; *P =* 0.0006), suggesting that an activated UPR may act as a protective mechanism against intrinsic and external stress in ALDH^+^ CSCs.

To study the specific function of IRE1α in stemness, we used the small molecule STF-083010, which specifically inhibits the endonuclease activity of IRE1α without affecting its other functions (33). Treatment with STF-083010 inhibited the splicing of *XBP1* at 10 μM and 25 μM in OVCAR5 and OVCAR3 cells, respectively (Supplemental Figure 8A). STF-083010 blocked spheroid formation by OC cell lines or by primary cells dissociated from HGSOC specimens (Figure 5, C and D), at doses lower than half of the established IC_50_ for these cells (Supplemental Figure 8, B and C). Additionally, STF-083010 decreased the percentages of ALDH^+^ cells within the treated cell populations (OVCAR5 *P =* 0.03; OVCAR3 *P =* 0.02; Figure 5E and Supplemental Figure 8D). Sorted ALDH^+^ cells from OVCAR5 were more sensitive to treatment with STF-083010 compared with ALDH^–^ cells, undergoing higher rates of apoptosis (Figure 6A, P *<* 0.0001). Treatment with STF-083010 also inhibited the expression of stemness-associated genes (*SOX2*, *OCT4*, *NANOG*, and *ALDH1A1*) and of IRE1α/XBP1s downstream genes (*XBP1s*, *HIF1α*, *VEFGA*, and *DDIT4*) in OVCAR5 (Figure 5, F and G) and OVCAR3 cells (Supplemental Figure 8E).

Further, we used *XBP1*-targeting shRNA (shXBP1) to examine the impact of XBP1 on stemness. XBP1 knockdown was confirmed by splicing assay, qPCR, and immunoblotting (Figure 6, B and C, and Supplemental Figure 8, F and H). Cells stably transduced with shXBP1 contained a significantly reduced ALDH^+^ population (Figure 6D and Supplemental Figure 8G) and had impaired spheroid-forming ability (Figure 5E and Supplemental Figure 8, I and J) compared with control cells. Additionally, the expression of stemness-associated genes (*SOX2*, *OCT4*, *NANOG*, and *ALDH1A1*) was reduced in cells transduced with shXBP1 compared with control (Figure 6F). These results support the notion that IRE1α-regulated XBP1 has an important functional role in CSCs.

### IRE1α is critical for FOXK2-mediated stemness in CSCs.

**Figure 7:**
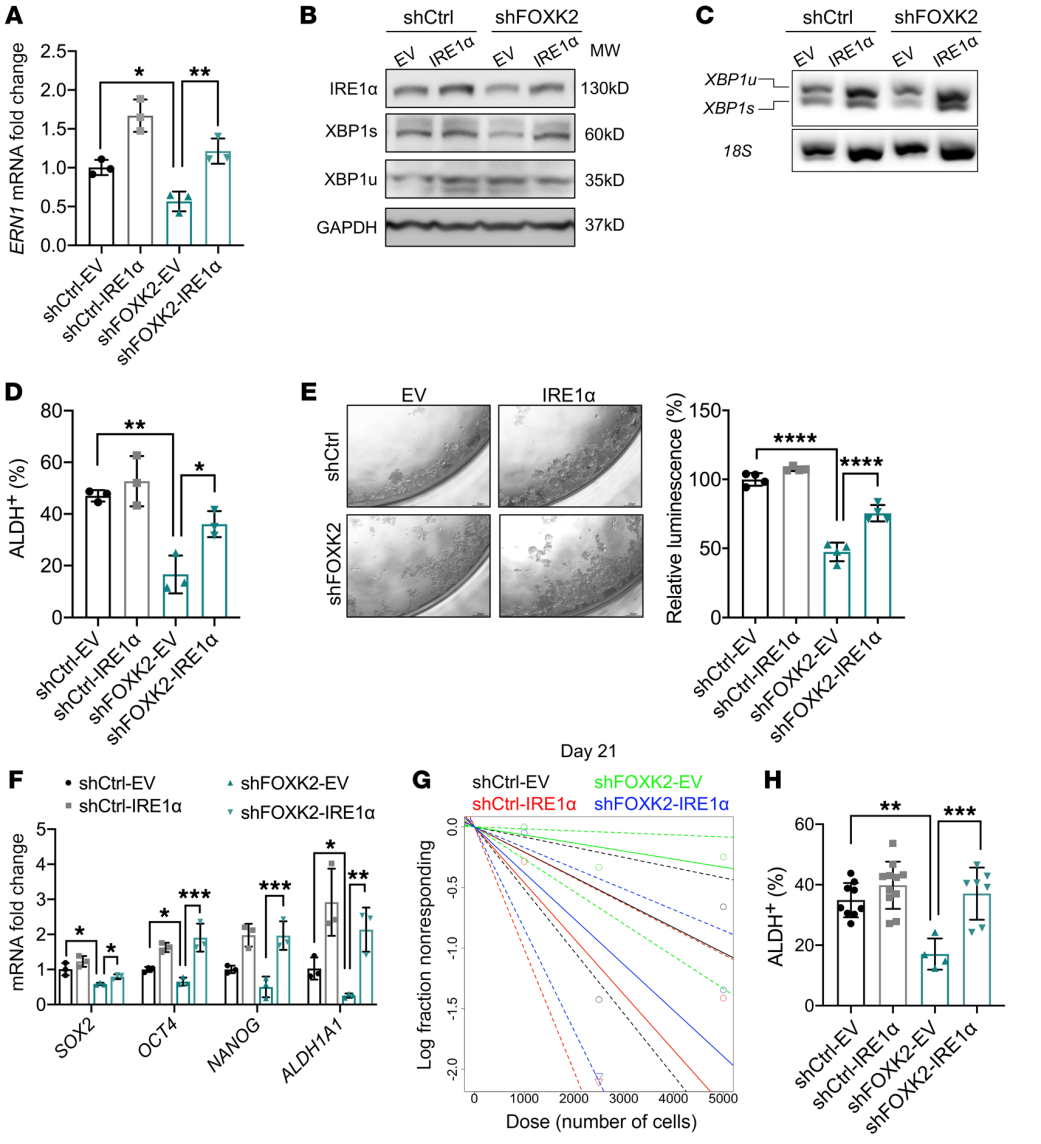
Effects of IRE1α rescue on stemness characteristics in FOXK2-deficient cells. (**A** and **B**) mRNA expression levels of *ERN1* measured by qRT-PCR (*n =* 3) (**A**), and protein levels (*n =* 3) by Western blotting of IRE1α, XBP1s, and XBP1u (**B**) in shCtrl and shFOXK2 OVCAR5 cells transfected with IRE1α-expressing plasmid (shCtrl-IRE1α, shFOXK2-IRE1α) or EV (shCtrl-EV, shFOXK2-EV). (**C**) RT-PCR–measured *XBP1* mRNA splicing (*XBP1u*, full-length transcript; *XBP1*s, spliced isoform) in shCtrl and shFOXK2 OVCAR5 cells transfected with IRE1α or EV. (**D**) Percentage of ALDH^+^ cells measured by flow cytometry (*n =* 3) in shCtrl and shFOXK2 OVCAR5 cells transduced with IRE1α or EV. (**E**) Pictures of spheroids (left) and spheroid formation (*n =* 4) assessed by cell viability assay (right) in shCtrl and shFOXK2 OVCAR5 cells transfected with EV or IRE1α (original magnification, ×20). (**F**) qRT-PCR–measured mRNA expression levels (*n =* 3) of stemness genes (*SOX2*, *OCT4*, *NANOG*) and CSC marker *ALDH1A1* in shCtrl and shFOXK2 OVCAR5 cells transfected with IRE1α (shCtrl-IRE1α, shFOXK2-IRE1α) or EV (shCtrl-EV, shFOXK2-EV). (**G**) Log-fraction plot of xenografts formed by the indicated numbers of shCtrl and shFOXK2 cells transduced with EV or IRE1α (*n =* 12) generated from ELDA. (**H**) ALDH^+^ CSC percentages among cells dissociated from xenografts derived from shCtrl and shFOXK2 cells transduced with EV or IRE1α. *P < 0.05; **P < 0.01; ***P < 0.005; ****P < 0.0001, by unpaired, 2-tailed Student’s *t* test when comparing 2 groups and 2-way ANOVA with Tukey’s multiple comparisons test when comparing more than 2 groups.

**Figure 8:**
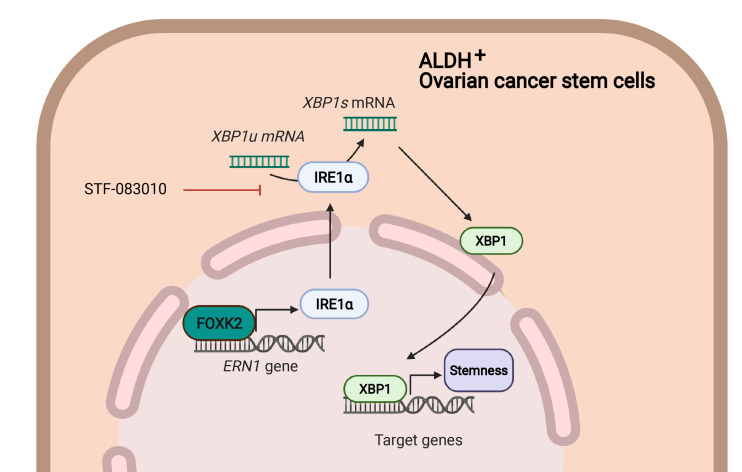
The proposed mechanism by which FOXK2 regulates the UPR and stemness. FOXK2 binds to the *ERN1* gene and activates its transcription. Increased IRE1α promotes *XBP1* mRNA splicing and enhances the stemness properties of ALDH^+^ cells. Illustration was created in BioRender (https://biorender.com/).

To determine whether IRE1α is the major downstream target of FOXK2 involved in stemness, we rescued its expression in FOXK2-depleted (shRNA knockdown) OVCAR5 and OVCAR3 cells. Restoration of IRE1α expression at mRNA (Figure 7A and Supplemental Figure 9A) and protein levels (Figure 7B and Supplemental Figure 9B) was confirmed in OVCAR5 and OVCAR3 cells. *XBP1s* mRNA levels were decreased in shFOXK2 OC cells transfected with EV (shFOXK2-EV) and restored in shFOXK2 cells transfected with IRE1α (shFOXK2-IRE1α) (Figure 7C). Further, the ALDH^+^ population, which was decreased in shFOXK2-EV cells, was rescued in shFOXK2-IRE1α to levels comparable to those observed in shRNA control cells transfected with EV (shCtrl-EV) (Figure 7D and Supplemental Figure 9C). Spheroid-forming ability was also increased in shFOXK2-IRE1α cells compared with shFOXK2-EV cells (Figure 7E and Supplemental Figure 9D), supporting the idea that restoration of IRE1α activity partially rescues inhibition of stemness imparted by FOXK2 knockdown. The expression of stemness genes (*SOX2*, *OCT4*, and *NANOG*) and *ALDH1A1* was partially rescued in shFOXK2-IRE1α compared with shFOXK2-EV cells (Figure 7F and Supplemental Figure 9E). To verify whether IRE1α overexpression in shFOXK2 cells rescues tumorigenicity, an in vivo serial dilution assay was performed, whereby shCtrl and shFOXK2 cells overexpressing EV or IRE1α were implanted in immunodeficient mice. Overexpression of IRE1α restored TIC in OVCAR5 cells transduced with shFOXK2. At 21 days, 7 of 12 tumors formed in the shFOXK2-IRE1α group compared with 1 of 12 tumors formed in the shFOXK2-EV group (*P =* 0.0094; Figure 7G and Supplemental Table 9). ELDA calculations also indicate that shFOXK2-IRE1α cells had increased estimated CSC frequency compared with shFOXK2-EV cells (1:2449 vs. 1:15,039, *P =* 0.00375; Supplemental Table 10). The ALDH^+^ CSC population was also rescued among cells dissociated from xenografts formed by shFOXK2-IRE1α compared with shFOXK2-EV cells (Figure 7H). Together, the data support the idea that IRE1α functions as a key FOXK2 target gene directly linked to maintenance of cancer stemness though tight regulation of protein homeostasis and response to cellular stress, as illustrated in the model in Figure 8.

## Discussion

In this study, we identified the TF FOXK2 as an active TF in ovarian CSCs implicated in regulation of cellular stress response. This proposed function of FOXK2 is based on several observations. First, we show that the *FOXK2* gene is upregulated in ovarian CSCs and in human ovarian tumors and has a critical role in regulating stemness properties, including TIC. Second, we demonstrate that FOXK2 fine tunes the UPR by regulating the transcription of *ERN1*, which then promotes splicing of *XBP1*. Third, by using a small molecule inhibitor of IRE1α (the protein product of the *ERN1* gene) and shRNA constructs targeting *XBP1*, the direct target of IRE1α, we provide evidence that disruption of these regulatory proteins, and hence the UPR, blocks proliferation of CSCs and stemness traits.

First, our results support the idea that FOXK2 expression is increased in CSCs versus non-CSCs and is associated with markers of stemness and with clinical outcomes in OC. Genetic manipulation of FOXK2 expression blocked stemness characteristics, including TIC, demonstrating a strong connection with the functions of CSCs. To our knowledge, FOXK2 association with cancer stemness had not been described and the functions of this TF in cancer are not well understood. Other members of the FOX family of TFs such as FOXA1, FOXM1, and FOXP1 are considered oncogenes (34) and FOXC1 was implicated in cancer stemness through modulation of β-catenin signaling (35). FOXK1, its paralog, has been linked to the progression of gastric (36), colorectal (37), and gallbladder cancer (38), and the 2 TFs share structural homology and certain functions (39, 40). Limited previous studies indicated that FOXK2 could act either as an oncogene or as a tumor suppressor, depending on context. For instance, FOXK1 and FOXK2 were shown to induce nuclear translocation of DVL and to activate WNT/β-catenin signaling in colorectal cancer (13). FOXK2 increased cell proliferation and migration by activating the PI3K/AKT pathway in hepatocellular carcinoma (41). On the other hand, FOXK2 was shown to act as a tumor suppressor in estrogen receptor–positive breast cancer by interacting with multiple corepressor complexes, causing suppression of cell proliferation and metastasis (14). Like other FOX TFs, FOXK1 and -2 have been linked to metabolic reprograming by activating several steps of aerobic glycolysis (15). In muscle cells and fibroblasts, FOXK1 and -2 repress starvation-induced autophagy through recruitment of Sin3A-HDAC complexes, which suppress expression of critical autophagy genes, counterbalancing the autophagy-activating effect of FOXO3 (16). FOXK1 and -2 were also shown to translocate to the nucleus following insulin stimulation and to induce transcription of genes involved in lipid metabolism (42).

Here, we demonstrate a previously unappreciated role of FOXK2. Our experiments uncovered that the UPR sensor IRE1α (*ERN1*) is a direct target of FOXK2. Although the interaction of FOXK2 with this specific intronic sequence of the *ERN1* gene had been detected in other cellular contexts (Supplemental Figure 5C), its functional significance was not recognized. Here we show that the binding of FOXK2 to this region located distally from the TSS, within intron 2 of the *ERN1* gene, was associated with deposition of the active mark H3K27Ac and had profound effects on transcription of *ERN1*. These effects were lost when FOXK2 binding to this region was blocked by catalytically inactive CRISPR-dCas9, supporting the notion that FOXK2 binding to this distal enhancer is critical for *ERN1* transcription. The functional consequences of this interaction implicate FOXK2 in the control of protein quality and cellular homeostasis.

Our data also support the idea that CSCs display and depend on the IRE1α/XBP1 system to preserve their stemness traits. Upon induction of ER stress, the UPR sensor protein IRE1α splices the mRNA encoding the transcription factor XBP1, leading to cytoprotective effects to prevent ER stress–induced apoptosis (43). In cancer cells, the UPR pathway is activated even in the absence of an obvious stress inducer, probably because cancer cells commonly undergo metabolic stress due to accelerated cell proliferation and increased requirements for protein and nucleotide synthesis (21). Reliance on a highly active sensor of the UPR may render CSCs to be fitter for survival in the presence of stress or exposure to cytotoxic drugs compared with non-CSCs. Indeed, in our models, CSCs were more sensitive to pharmacological inhibition of IRE1α or to XBP1 knockdown compared with non-CSCs. Our findings are consistent with recent results in other cancers. In preleukemic stem cells, oncogenic N-Ras^G12D^ was shown to activate the IRE1α/XBP1 axis to promote cell survival (44). *XBP1* mRNA splicing was found to be increased in CD44^hi^CD24^lo^ populations in triple-negative breast cancer, a stem-like cell population (20). In that study, XBP1s was shown to contribute to tumor progression by activating HIF-1α (20), a target we also found to be upregulated by FOXK2, downstream of IRE1α.

Other studies have reported an association between the IRE1α/XBP1 pathway and MYC signaling, a transcription factor widely associated with stemness, cell self-renewal, and chemoresistance (45–47). In breast cancer, MYC was shown to directly regulate the transcription of *ERN1* by binding to its promoter and enhancer and to cooperate with XBP1, leading to enhanced transcriptional activity (48). MYC-driven cancer cells were found to be highly dependent on IRE1α/XBP1 and exquisitely sensitive to pharmacological inhibition of IRE1α (48, 49). Conversely, a small molecule inhibitor targeting IRE1α was shown to decrease c-MYC levels and inhibit growth of prostate cancer cells and tumors (50), demonstrating a regulatory feedback loop between XBP1 and c-MYC. Our data demonstrate the direct significance of the IRE1α/XBP1 axis in ovarian CSCs. Blocking this pathway by using either genetic or pharmacological strategies potently inhibited the ALDH^+^ population and spheroid formation in several OC models. The increased levels of XBP1s in CSCs may promote stemness either through enhanced HIF-1α or c-Myc signaling, as shown in other systems, or through other, yet uncovered targets.

In conclusion, we have unveiled key functions of FOXK2 in the regulation of the UPR in cancer cells and CSCs. Our findings have implications for designing novel strategies to target this recalcitrant cell population either by inhibiting the regulatory functions of FOXK2 or by modulating UPR signaling.

## Methods

### Cell culture and treatment.

OVCAR5 cells were provided by Marcus Peter at Northwestern University (Chicago, Illinois, USA). OVCAR3, CAOV3, and OV90 cells were purchased from ATCC. COV362, Kuramochi, OVCAR4, and OVCAR8 cells were provided by Kenneth Nephew at Indiana University (Bloomington, Indiana, USA). NoEM cells were provided by Serdar Bulun at Northwestern University (Chicago, Illinois, USA). Immortalized human FTE cells (FT190) were from Ronny Drapkin at the University of Pennsylvania (Philadelphia, Pennsylvania, USA). Cells were maintained in a 37°C incubator with 5% CO_2_, and cell culture conditions are shown in Supplemental Table 11. All experiments were performed using low-passage cells. Cell lines were confirmed to be pathogen- and mycoplasma-negative by Charles River Animal Diagnostic Services and were also periodically tested by Universal Mycoplasma Detection Kit (ATCC). The IRE1α inhibitor STF-083010 was obtained from Sigma-Aldrich. Cells were treated with indicated doses every 2 days and collected on day 5 for experiments. The ER stress–inducing agent tunicamycin was obtained from Cayman Chemical Company.

### Human specimens.

HGSOC tumors or associated malignant ascites (*n =* 7) were collected and dissociated immediately into single-cell suspensions. Briefly, the HGSOC tumors were minced into small pieces and digested with collagenase I (Sigma-Aldrich) and hyaluronidase (Sigma-Aldrich) at 37°C for 3 hours. Cells were filtered, washed with PBS, and treated with red blood cell lysis buffer (Sigma-Aldrich) and DNase I (Sigma-Aldrich) for purification, as previously described (22, 51). A tissue microarray (TMA) was built from deidentified HGSOC specimens (*n =* 23) from patients who had undergone 3 to 6 cycles of platinum-taxane NACT (IRB-approved CSR protocol 1247). Each specimen was entered in duplicate and FTE (*n =* 6) served as controls. Patients’ characteristics were previously described (22).

### Flow cytometry analysis and FACS.

ALDH^+^ and ALDH^–^ cells were identified by using an ALDEFLUOR kit (Stem Cell Technologies) by following the ALDEFLUOR protocol. Briefly, cells were suspended in ALDEFLUOR assay buffer with addition of 1.5 mM ALDH substrate and incubated at 37°C for 40 minutes. ALDH substrate was washed away with cold ALDEFLUOR buffer before analysis. Diethylaminobenzaldehyde (DEAB), which is a specific inhibitor of ALDH, was used to control for background fluorescence. Cells were analyzed by LSR Fortessa flow cytometer (BD) and sorted with a FACSAria 6-laser sorter (BD). For apoptosis analysis, cells were stained with APC Annexin V (BioLegend) by following the manufacturer’s protocol and analyzed by LSR Fortessa flow cytometer.

### Lentiviral knockdown system.

Lentiviral transduction particles containing shRNAs were used for generating stable-knockdown cell lines. shFOXK2 (SHCLNV-NM_004514, Sigma-Aldrich) and shXBP1 (SHCLNV-NM_005080, Sigma-Aldrich) were transduced following the manufacturer’s protocol with 8 μg/mL Polybrene (Thermo Fisher Scientific) added to the media. The shRNA sequences are included in Supplemental Table 12. pLKO.1-puro nontargeting shRNA was used as control (shCtrl). Stable cells were selected with puromycin (2 μg/mL for OVCAR5 and COV362; 1 μg/mL for OVCAR3) starting 48 hours after transduction. Knockdown efficiency was assessed by qPCR assay.

### Plasmid construction and establishment of overexpressing cells.

The FOXK2-expressing vector [FOXK2 cDNA ORF-pcDNA3.1/C(K)DYK, OHu30465, Genscript] was used for generating FOXK2-OE cell lines. The IRE1α-expressing vector was used for generating rescue overexpression in FOXK2-knockdown cells. The IRE1α-expressing vector was constructed by amplifying the full-length cDNA encoding IRE1α from the IRE1α-pcDNA3.EGFP plasmid (13009, Addgene) and ligating it into the pcDNA3.1 vector. Empty pcDNA3.1 vector (Invitrogen) was used as control. Sequences of plasmid were verified by Sanger sequencing. OC cells (OVCAR5 and OVCAR3) were transfected by using Lipofectamine 2000 (Invitrogen). Forty-eight hours after transfection, G418 sulfate was added to the complete culture medium (200 μg/mL for OVCAR5, 400 μg/mL for OVCAR3) to establish stably transfected cells. To rescue FOXK2 expression, cDNA encoding Foxk2 was amplified from the *Foxk2* (NM_001080932) Mouse Tagged ORF Clone (ORIGENE) and subcloned into the pLenti-CMV vector. Empty pLenti-CMV vector was used as a control. HEK-293T cells were cotransfected with pLenti-Foxk2 and packaging mix using Lipofectamine 2000 reagent to generate lentiviral particles for transduction, as described above.

### qRT-PCR analysis and XBP1 splicing assay.

RNA was purified by using TRI Reagent (Sigma-Aldrich) or by using the RNeasy Micro kit (QIAGEN). For each sample, 1 μg of total was reverse transcribed (Applied Biosystems) and analyzed by SYBR Green–based real-time PCR (Applied Biosystems). Primers are shown in Supplemental Table 13. *18S* rRNA was used as control. The *XBP1* splicing assay was performed as previously described (52). Briefly, 5 ng cDNA was amplified by PCR with GoTaq Green Master Mix (Promega) and *XBP1* primers. PCR products were visualized by electrophoresis in 2.5% agarose gels (Invitrogen).

### In vitro tumor spheroid-forming assay.

OC cells were seeded at 500 cells per well into 96-well ultra-low attachment plates (Corning) and cultured in MammoCult medium (Stem Cell Technologies) supplemented with 4 μg/mL heparin and 0.48 μg/mL hydrocortisone (Stem Cell Technologies). After 14 days, tumor spheroids were visualized by inverted microscope (×4 magnification). Cell viability was assessed by CellTiter-Glo 3D cell viability assay (Promega).

### In vivo xenograft experiments and ELDA.

Female athymic nude mice (8 weeks old, Envigo) were injected with serially diluted shCtrl- and shFOXK2-transduced OVCAR5 cells mixed with an equal volume of Matrigel (Corning). After tumor inoculation, mouse weight and tumor growth were monitored and recorded twice per week. Tumor dimensions (length, width, and depth) were measured with a digital caliper, and tumor volumes were calculated as volume = 0.5 × length × width × depth. Mice were euthanized and tumors were harvested on day 32 after inoculation of tumor cells for shCtrl versus shFOXK2 experiments, day 45 for Foxk2 rescue experiments, and day 41 for IRE1α rescue experiments. Tumors were dissociated into single cells for subsequent analysis. The CSC frequency was calculated by using ELDA software (http://bioinf.wehi.edu.au/software/elda/), including estimated frequency, confidence interval, and statistical significance (53). Tumorigenicity was analyzed by χ^2^ test.

### RNA-seq and data analysis.

For each sample, 1 μg of total input RNA was extracted by TRI Reagent, and DNA was removed by using an RNeasy MinElute Cleanup Kit (QIAGEN) with RNase-Free DNase Set (QIAGEN). mRNA was isolated by NEBNext Poly(A) mRNA Magnetic Isolation Module, and RNA sequencing libraries was prepared using NEBNext Ultra II RNA Library Prep Kit following the manufacturer’s recommendations (New England Biolabs). Library qualities were evaluated by High Sensitivity DNA Assay (Agilent Technologies) and sequenced by Illumina HiSeq 4000 sequencer (single-end 50 bp). Raw sequence generated from HiSeq 4000 was converted into fastq files and demultiplexed with bcl2fastq software v2.17.1.14 (https://support.illumina.com/sequencing/sequencing_software/bcl2fastq-conversion-software/downloads.html). After checking quality with the FastQC tool, raw sequencing reads were aligned to human genome build hg38 using STAR v.2.5.2 (https://github.com/alexdobin/STAR) with standard settings. Mapped reads were converted to raw counts with HTSeq (https://htseq.readthedocs.io/en/master/), normalized to library size, and analyzed for DEGs by edgeR (Bioconductor). The log_2_(fold change) and *P* value of total normalized counts and DEG counts were then analyzed by GSEA and Ingenuity Pathway Analysis (IPA, QIAGEN) with standard settings. Data are deposited in the NCBI gene expression omnibus (GEO GSE173779).

### TCGA and GTEx data analysis and survival analysis.

The expression levels of genes (*FOXK2*, *ERN1)* in OC specimens (*n =* 427) were obtained from the RNA-seq data set profiled by TCGA and the expression of *FOXK2* in normal FTE specimens (*n =* 5) was obtained from the GTEx project. All data were downloaded from the UCSC Xena browser (https://xenabrowser.net/) as RSEM counts. The Kaplan-Meier survival curves were plotted with an online tool using microarray data from GEO and TCGA (*n =* 614; ref. 54). The statistical significance of survival differences between groups with high/low level of expression was determined by using the log-rank test.

### ChIP.

ChIP was performed with anti-FOXK2 and anti-H3K27Ac antibodies (Supplemental Table 14). Briefly, extracted chromatin was crosslinked with 1% paraformaldehyde and fragmented to an average size of approximately 300–500 bp by sonication. Chromatin (10 μg) was incubated with 5 μg of either anti-FOXK2 or anti-H3K27Ac antibody for immunoprecipitation. The concentration of immunoprecipitated DNA was measured with a Qubit dsDNA HS Assay Kit (Thermo Fisher Scientific). For ChIP-qPCR analysis, immunoprecipitated DNA was amplified by qPCR with gene-specific primers (Supplemental Table 15) using SYBR Green Master Mix (Bio-Rad). Input DNA was used for normalization and a target sequence located 1 kb from the binding site was used as control.

### ChIP-seq and data analysis.

ChIP-seq libraries were prepared using a KAPA Hyper Prep kit (Roche Sequencing) with KAPA UDI Adaptor (Roche Sequencing). Qualities of libraries were checked by High Sensitivity DNA Assay (Agilent Technologies) and sequenced on an Illumina HiSeq 4000 sequencer. Raw sequencing data generated from Illumina HiSeq was converted into fastq files, which were checked by FastQC (https://github.com/s-andrews/FastQC). Raw single-end 50-bp sequencing reads were mapped to human genome build hg38 using Bowtie2 v.2.2.6 (http://bowtie-bio.sourceforge.net/bowtie2/index.shtml) with standard settings. FOXK2 ChIP-seq peaks were identified by HOMER v.4.10 (http://homer.ucsd.edu/homer/). Two biological replicates were compared. Normalized read counts and differential enrichment statistics for each peak were analyzed by DESeq2 Software (Bioconductor). Motif analysis was performed by HOMER (v.4.10). Data are deposited in the NCBI GEO (GSE173777). The OVCAR5 cell H3K27Ac ChIP-seq results were generated by reanalyzing published data (24) (GEO GSM4271303; OVCAR5 H3K27Ac, SRR10887072; input, SRR10887252).

### CRISPR-dCas9 vector construction and lentiviral production.

The CRISPR-dCas9-ERN1 vectors were constructed by using lentiCRISPRv2-dCas9, a lentiviral plasmid for expression of gRNA and dCas9 that is derived from lentiCRISPR v2 (112233, Addgene; ref. 55). gRNAs were designed using the web-based gRNA design tool CRISPOR (http://crispor.org) (56), and the target sequences are shown in Supplemental Table 16. gRNAs were ligated to the CRISPR-dCas9 backbone by following a protocol modified from lentiCRISPR v2 (57). Three gRNAs were selected based on the location of the FOXK2 binding region (chr17: 64,081,703–64,083,050) along with a nontargeting control guide (NT). Insertion of gRNAs was verified by Sanger sequencing. The lentivirus was produced by cotransfecting lentiCRISPR-dCas9, psPAX2 (12260, Addgene), and pMD2.G (12259, Addgene) at a 1:4:2 ratio into HEK-293T cells. The virus-containing supernatant was filtered through a 0.45-μm PES filter (MilliporeSigma) before being added to OVCAR5 cells.

### IHC.

Sections (5 μm) of paraffin-embedded xenograft tissues or OC tissue microarrays were heated at 56°C for 20 minutes and deparaffinized. The slides were processed for heat-induced epitope retrieval and incubated with anti-FOXK2 antibody (dilution 1:100; Supplemental Table 14). Rabbit IgG was used as negative control (Santa Cruz Biotechnology). Slides were blocked with Dako Biotin Blocking System (Agilent) and color was developed by using Dako DAB+ Substrate Chromogen System (Agilent). Slides were imaged with TissueFAXS PLUS (TissueGnostics) or DFC295 (Leica).

### Immunoblot analysis.

Cells were harvested and lysed using RIPA buffer containing Halt Protease and Phosphatase Inhibitor (Thermo Fisher Scientific). Protein lysate was quantified using Protein Assay (Bio-Rad) and resolved by using 10% SDS-PAGE and transferred to PVDF membrane. Primary antibodies (1:1000) used for immunoblotting analysis are shown in Supplemental Table 14. Blots were then incubated with HRP-conjugated secondary (1:2500) anti-rabbit antibody (Cytiva Lifescience) or anti-mouse antibody (R&D Systems) and visualized by SuperSignal West Femto Maximum Sensitivity Substrate (Thermo Fisher Scientific).

### Data availability.

All high-throughput sequencing data and processed data have been deposited in the NCBI GEO data repository: GSE173780. The analysis was performed by using publicly available software described in the Methods.

### Statistics.

Data are presented as mean ± SD. Statistical significance was determined by using 2-tailed Student’s *t* test when comparing 2 groups and 2-way ANOVA with Tukey’s multiple comparisons test when comparing more than 2 groups (Prism 8, GraphPad Software). ELDA and tumorigenicity were analyzed by χ^2^ test. *P* values of less than 0.05 were considered statistically significant, with *P < 0.05, **P < 0.01, ***P < 0.005, ****P < 0.001 indicated in the figures. The number of biological replicates in each panel is indicated by *n*.

### Study approval.

HGSOC tumors or associated malignant ascites were collected fresh under Northwestern University–approved protocol (IRB STU#00202468). Animal studies were approved by the Northwestern University Institutional Animal Care and Use Committee (IACUC, protocol IS00003060).

## Author contributions

YZ, YW, and DM conceptualized the project and research plan. YZ and YW designed experiments and performed assays. EJT collected tissue samples for the study. YZ and GZ designed the bioinformatic analysis pipeline and wrote the code for the bioinformatic analysis pipeline. YZ analyzed data and wrote the initial manuscript. DM and MA revised the manuscript. DM supervised the project and acquired funding.

## Supplementary Material

Supplemental data

## Version 1 03/29/2022

In-Press Preview

## Version 2 05/16/2022

Electronic publication
