# Supplementary material for: FOXK2 promotes ovarian cancer stemness by regulating the unfolded protein response pathway
Source: J Clin Invest. 2022 May 16;132(10):e151591. doi: 10.1172/JCI151591 (PMC9106354; doi:10.1172/JCI151591)
Supplement: Supplemental data [file jci-132-151591-s168.pdf]

Supplementary Information for:

FOXK2 Promotes Ovarian Cancer Stemness by Regulating the Unfolded Protein Response Pathway

Yaqi Zhang, Yinu Wang, Guangyuan Zhao, Edward J. Tanner, Mazhar Adli, Daniela Matei

Table of contents:

1. Supplementary Figures
2. Supplementary Tables

Supplementary Materials 1
Supplementary Figures

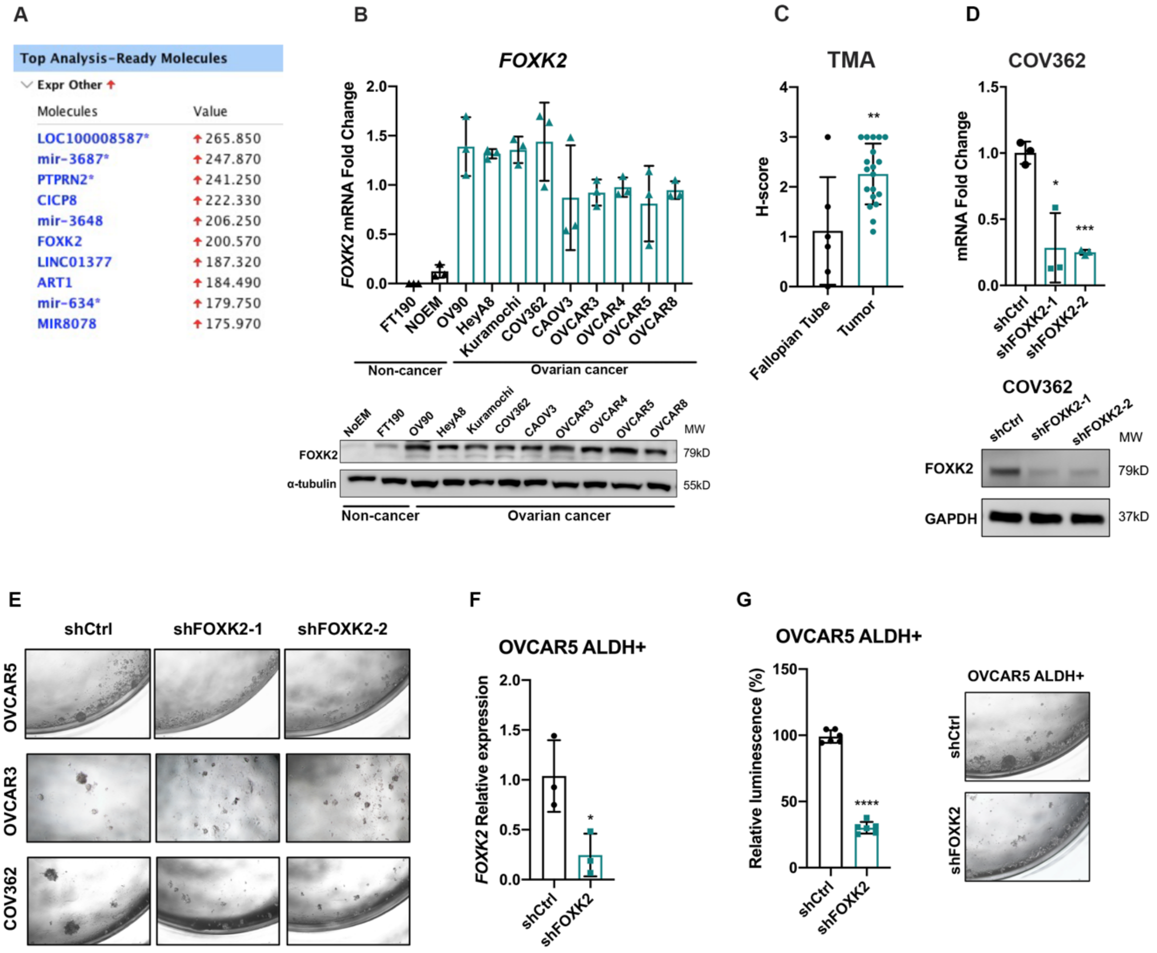

Supplementary Figure 1. FOXK2 expression is upregulated in OC cells and tumors and is associated with stemness

A, Top 10 genes associated with open chromatin state determined by ATAC-seq in $ALDH^+$ $CD133^+$ CSCs vs. ALDH-CD133- non-CSC cells sorted by FACS from OVCAR5 cells. **B,** Expression of *FOXK2* mRNA (n = 3) quantified by qRT-PCR (top panel), and FOXK2 protein levels measured by western blotting (bottom panel) in fallopian tube epithelium (FT190) and normal endometrium (NoEM), and in nine OC cell lines (OV90, HeyA8, Kuramochi, COV362, CAOV3, OVCAR3, OVCAR4, OVCAR5 and OVCAR8). Alpha-tubulin was the loading control. **C,** H-score quantifies expression of FOXK2 measured by IHC in sections of fallopian tube (n = 6) and tumors residual post neoadjuvant chemotherapy (n = 19) on a tissue microarray (TMA) (p = 0.0031, tumors vs. fallopian tube). **D,** *FOXK2* mRNA expression measured by qRT-PCR (n = 3, top panel) and FOXK2 protein (western blot, bottom panel) in COV362 cells transduced with two shRNAs targeting *FOXK2* (shFOXK2-1 and shFOXK2-2) or control shRNA (shCtrl). **E,** Representative images of spheroids (20X magnification) formed from shFOXK2 and shCtrl OVCAR5, OVCAR3 and COV362 cells (n = 6 per cell line). **F,** *FOXK2* mRNA expression in sorted ALDH+ OVCAR5 cells transduced with shRNA targeting FOXK2 (shFOXK2) and non-targeting shRNA (shCtrl) measured by qRT-PCR (n = 3). **G,** Representative images of spheroids (20X magnification), and quantification of cell viability in spheroids (n = 6) formed from sorted ALDH+ OVCAR5 cells transduced with shFOXK2 and shCtrl. For all panels: * $p<0.05$, ** $p<0.01$, *** $p<0.005$, **** $p<0.0001$. Data were analyzed by unpaired two-tailed Student's t test.

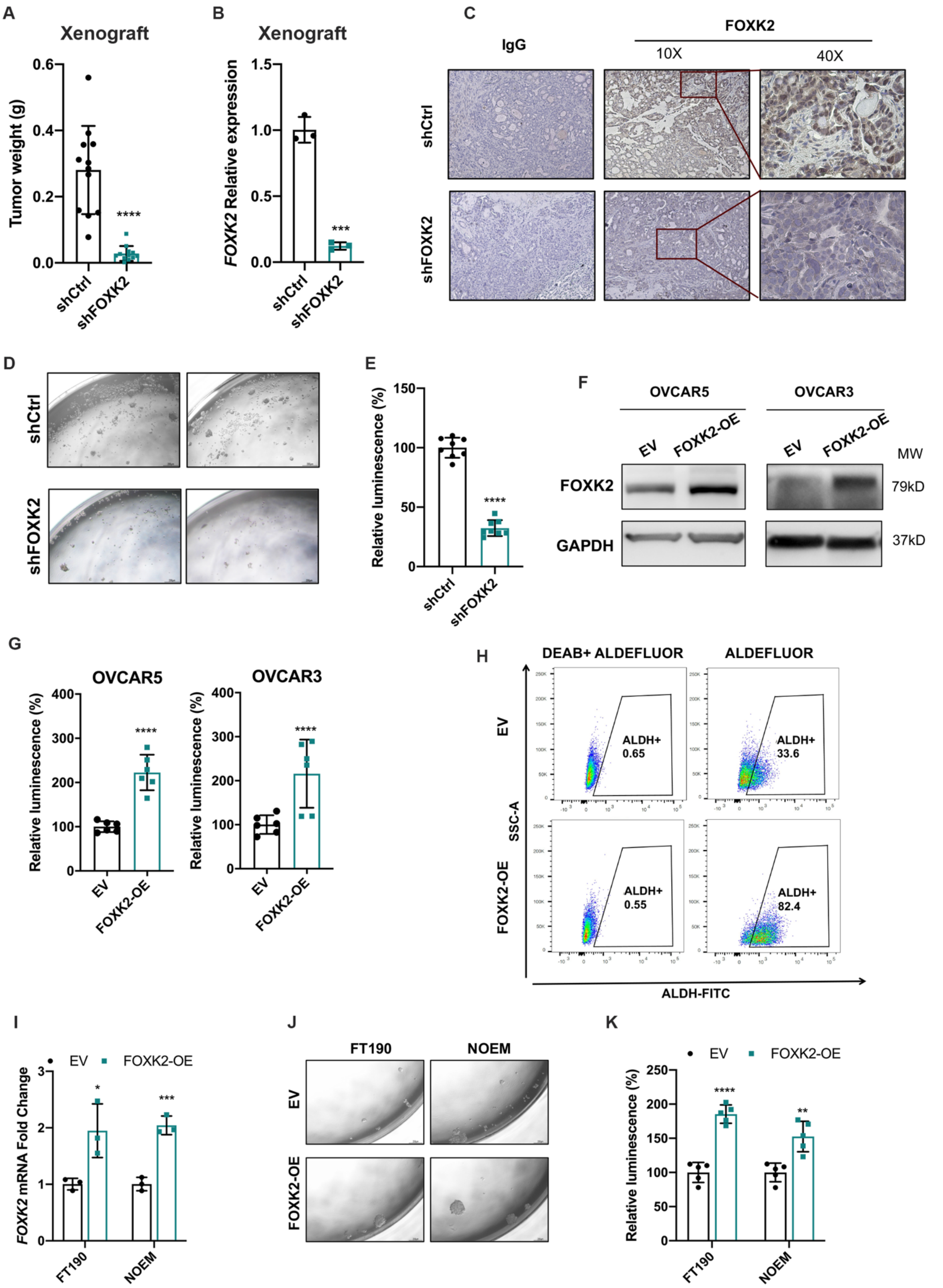
A
Xenograft
Tumor weight (g)
shCtrl
shFOXK2
****
B
Xenograft
FOXK2 Relative expression
***
C
IgG
FOXK2
10X
40X
shCtrl
shFOXK2
D
shCtrl
shFOXK2
E
Relative luminescence (%)
****
F
OVCAR5
OVCAR3
EV
FOXK2-OE
MW
FOXK2
79kD
GAPDH
37kD
G
OVCAR5
OVCAR3
Relative luminescence (%)
****
H
DEAB+ ALDEFLUOR
ALDEFLUOR
EV
FOXK2-OE
SSC-A
ALDH+ 0.65
ALDH+ 33.6
ALDH+ 0.55
ALDH+ 82.4
ALDH-FITC
I
EV
FOXK2-OE
FOXK2 mRNA Fold Change
FT190
NOEM
J
FT190
NOEM
EV
FOXK2-OE
K
EV
FOXK2-OE
Relative luminescence (%)
FT190
NOEM

Supplementary Figure 2. FOXK2 regulates stemness in OC models

A, Weight of tumor xenografts from mice injected with 2,500, 5,000, or 10,000 shCtrl and shFOXK2 transduced OVCAR5 cells/site (n = 12 mice). **B, C,** Expression of *FOXK2* mRNA measured by qRT-PCR (n = 3) (**B**) and by IHC (**C**) in a subset of tumor xenografts described in (**A**). **D,** Images of spheroids formed from cells dissociated from tumor xenografts induced by shCtrl and shFOXK2 transfected OVCAR5 cells described in (**A**). **E,** Quantification of cell viability measures spheroid abundance in cultures of cells dissociated from xenografts derived from shCtrl and shFOXK2 transduced OVCAR5 cells (n = 6). **F,** FOXK2 protein expression measured by western blotting in OVCAR5 and OVCAR3 cells transfected with empty vector (EV) or FOXK2 expression vector (FOXK2-OE). **G,** Viability of tumor spheroid (n = 6) formed from OVCAR5 and OVCAR3 cells transduced with EV or FOXK2-OE. **H,** Analysis of ALDH+ cells measured by flow cytometry in EV and FOXK2-OE OVCAR5 cells (n = 3). **I,** mRNA expression levels for *FOXK2* in FT190 and NOEM cells transduced with EV or FOXK2-OE. **J-K,** Representative images (20X) (**J**) and cell viability (**K**) of tumor spheroids formed from FT190 and NOEM cells transduced with EV or FOXK2 (n = 5 per cell line). For all panels: * $p<0.05$, ** $p<0.01$, *** $p<0.005$, **** $p<0.0001$. Data were analyzed by unpaired two-tailed Student's t test.

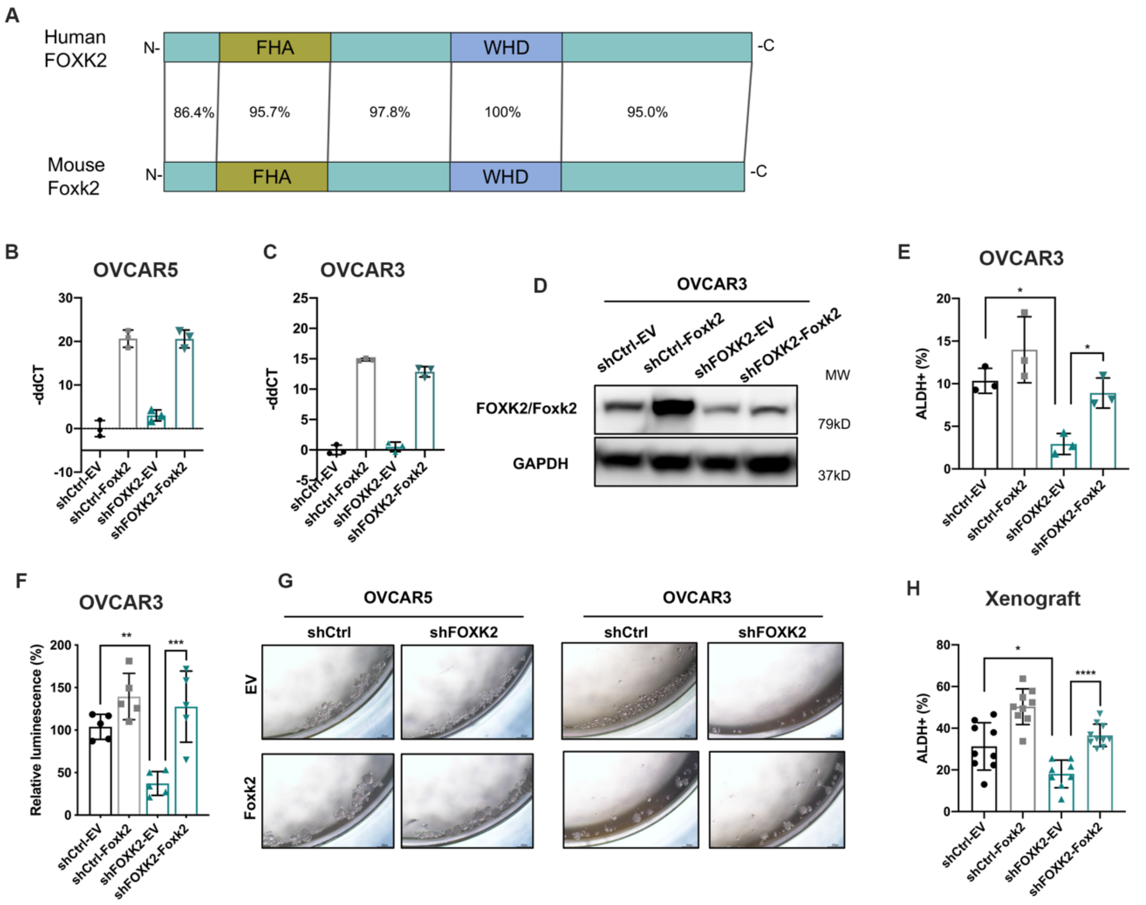

Supplementary Figure 3. Overexpression of mouse Foxk2 protein rescues the loss-of stemness in shFOXK2 cells

A, Comparison of human FOXK2 and mouse Foxk2 protein domains. **B-C,** mRNA expression levels of mouse *Foxk2* in OVCAR5 (**B**) and OVCAR3 (**C**) cells transduced with empty vector (EV) or Foxk2. **D,** Western-bolt images showing the levels of FOXK2/Foxk2 protein in OVCAR3 shCtrl and shFOXK2 cells transduced with EV (shCtrl-EV, shFOXK2-EV) or Foxk2 (shCtrl-Foxk2, shFOXK2-Foxk2) (n = 3). **E-F,** Percentage of ALDH+ CSCs (n = 3) (**E**) and cell viability in spheroid cultures (n = 6) (**F**) in OVCAR3 shCtrl and shFOXK2 cells transduced with EV or Foxk2. **G,** Representative images of spheroids (20X) formed from shCtrl and shFOXK2 cells transduced with EV or Foxk2 OVCAR5 and OVCAR3 cells (n = 6). **H,** Flow cytometry measures the ALDH+ CSCs population among cells dissociated from tumor xenografts harvested from the serial limiting dilution experiment described in Figure 2K (n = 8 for shFOXK2-EV, and n = 9 for other groups). For all panels: * $p<0.05$, ** $p<0.01$, *** $p<0.005$, **** $p<0.0001$. Data were analyzed by unpaired two-tailed Student's t test when comparing two groups and two-way ANOVA with multiple comparisons when comparing more than two groups.

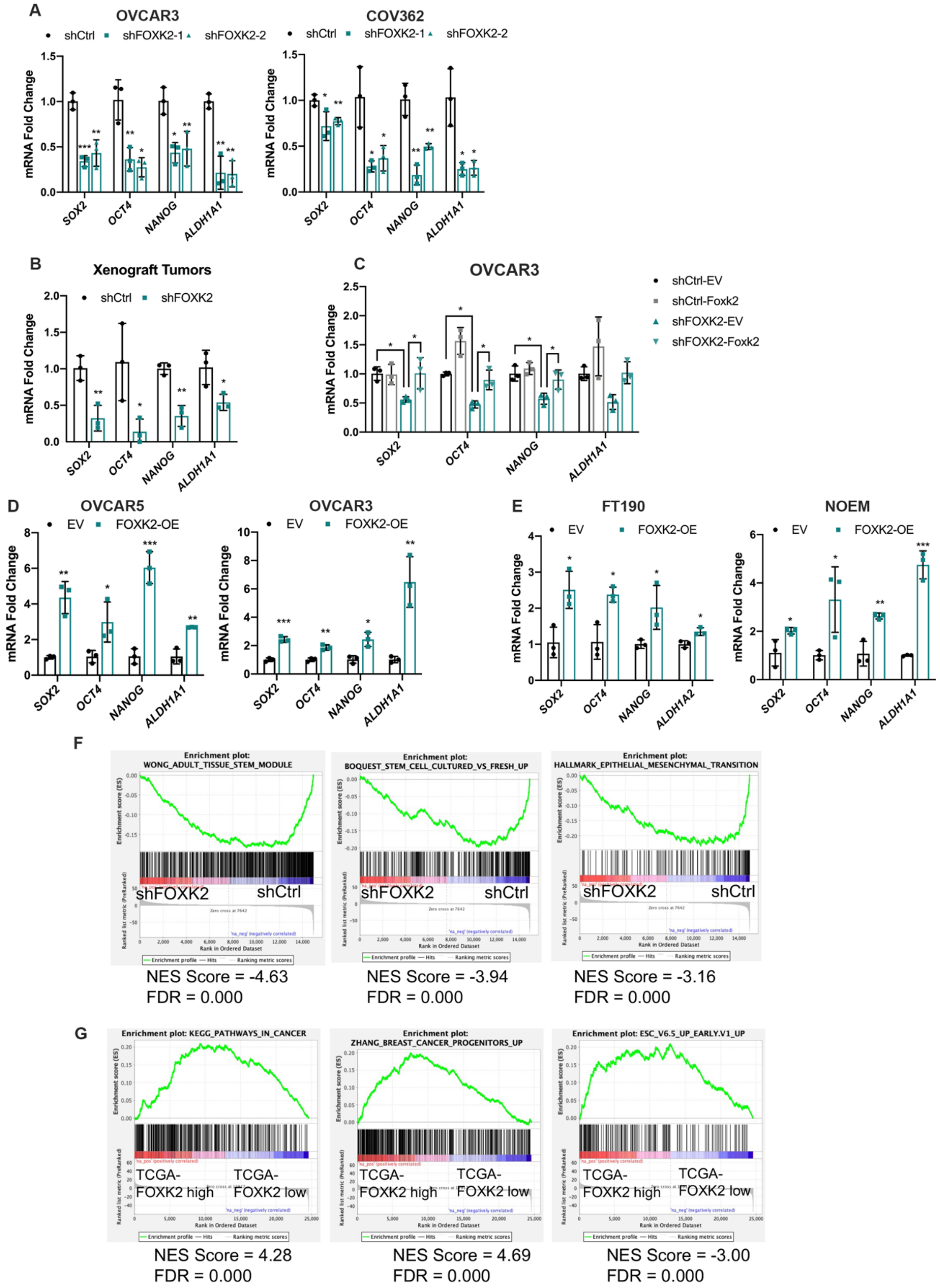
A
OVCAR3
shCtrl shFOXK2-1 shFOXK2-2
COV362
mRNA Fold Change
SOX2
OCT4
NANOG
ALDH1A1
B
Xenograft Tumors
shCtrl shFOXK2
C
OVCAR3
shCtrl-EV
shCtrl-Foxk2
shFOXK2-EV
shFOXK2-Foxk2
D
OVCAR5
EV FOXK2-OE
E
FT190
NOEM
ALDH1A2
F
Enrichment plot: WONG_ADULT_TISSUE_STEM_MODULE
Enrichment plot: BOQUEST_STEM_CELL_CULTURED_VS_FRESH_UP
Enrichment plot: HALLMARK_EPITHELIAL_MESENCHYMAL_TRANSITION
shFOXK2 shCtrl
NES Score = -4.63
FDR = 0.000
NES Score = -3.94
FDR = 0.000
NES Score = -3.16
FDR = 0.000
G
Enrichment plot: KEGG_PATHWAYS_IN_CANCER
Enrichment plot: ZHANG_BREAST_CANCER_PROGENITORS_UP
Enrichment plot: ESC_V6.5_UP_EARLY.V1_UP
TCGA-FOXK2 high
TCGA-FOXK2 low
NES Score = 4.28
FDR = 0.000
NES Score = 4.69
FDR = 0.000
NES Score = -3.00
FDR = 0.000

Supplementary Figure 4. Expression of FOXK2 is associated with stemness-associated gene sets

A-B, mRNA expression levels of stemness genes (*SOX2, OCT4, NANOG*) and *ALDH1A1* (n = 3) in OVCAR3 and COV362 cells transduced with non-targeting shRNA (shCtrl) or shRNAs targeting FOXK2 (shFOXK2-1 and shFOXK2-2) (**A**) and in xenografts derived from shCtrl and shFOXK2 OVCAR5 cells (**B**). **C,** mRNA expression levels for stemness associated genes (*SOX2, OCT4, NANOG*) and *ALDH1A1* (n = 3) in OVCAR3 shCtrl or shFOXK2 cells transduced with EV or Foxk2. **D,** mRNA expression levels (n = 3) of *SOX2, OCT4, NANOG* and *ALDH1A1* in OVCAR5 and OVCAR3 cells transduced with EV or FOXK2-OE. **E,** mRNA expression levels (n = 3) of *SOX2, OCT4, NANOG* and *ALDH1A1* in FT190 and NOEM cells transduced with EV or FOXK2-OE. **F,** Enrichment plots illustrate expression of genes included in the "*Wong adult tissue stem module*", "*cultured stem cells*", and "*epithelial-mesenchymal transition*" gene sets obtained by Gene Sets Enrichment Analysis (GSEA) of DEGs between shCtrl and shFOXK2 transduced OVCAR5 cells (RNA-seq). **G,** GSEA enrichment plots of "*KEGG pathways in cancer*", "*breast cancer progenitors*", and "*ESC early stage*" gene sets based on analysis of TCGA ovarian cancer samples with high versus low expression of FOXK2 (n = 427). For all panels: * $p<0.05$, ** $p<0.01$, *** $p<0.005$. Data were analyzed by unpaired two-tailed Student's t test.

A

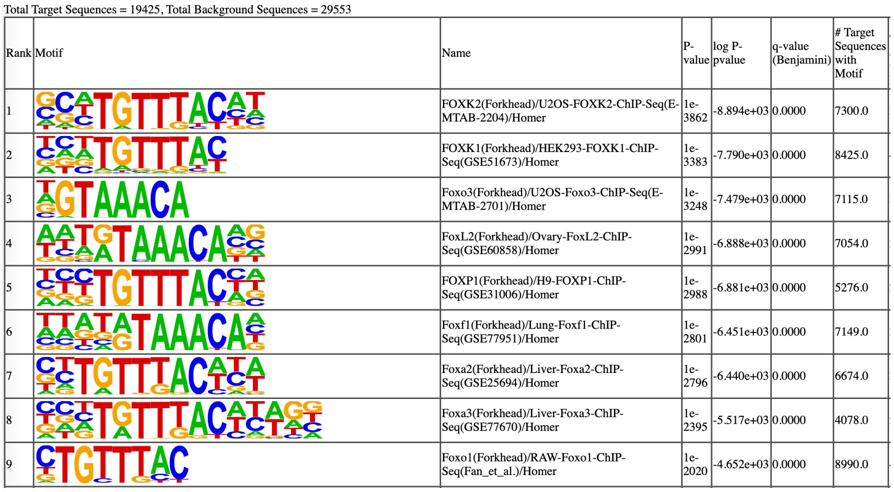

Total Target Sequences = 19425, Total Background Sequences = 29553

Rank	Motif	Name	P-value	log P-pvalue	q-value (Benjamini)	# Target Sequences with Motif
1		FOXK2(Forkhead)/U2OS-FOXK2-ChIP-Seq(E-MTAB-2204)/Homer	1e-3862	-8.894e+03	0.0000	7300.0
2		FOXK1(Forkhead)/HEK293-FOXK1-ChIP-Seq(GSE51673)/Homer	1e-3383	-7.790e+03	0.0000	8425.0
3		Foxo3(Forkhead)/U2OS-Foxo3-ChIP-Seq(E-MTAB-2701)/Homer	1e-3248	-7.479e+03	0.0000	7115.0
4		FoxL2(Forkhead)/Ovary-FoxL2-ChIP-Seq(GSE60858)/Homer	1e-2991	-6.888e+03	0.0000	7054.0
5		FOXP1(Forkhead)/H9-FOXP1-ChIP-Seq(GSE31006)/Homer	1e-2988	-6.881e+03	0.0000	5276.0
6		Foxf1(Forkhead)/Lung-Foxf1-ChIP-Seq(GSE77951)/Homer	1e-2801	-6.451e+03	0.0000	7149.0
7		Foxa2(Forkhead)/Liver-Foxa2-ChIP-Seq(GSE25694)/Homer	1e-2796	-6.440e+03	0.0000	6674.0
8		Foxa3(Forkhead)/Liver-Foxa3-ChIP-Seq(GSE77670)/Homer	1e-2395	-5.517e+03	0.0000	4078.0
9		Foxo1(Forkhead)/RAW-Foxo1-ChIP-Seq(Fan_et_al.)/Homer	1e-2020	-4.652e+03	0.0000	8990.0

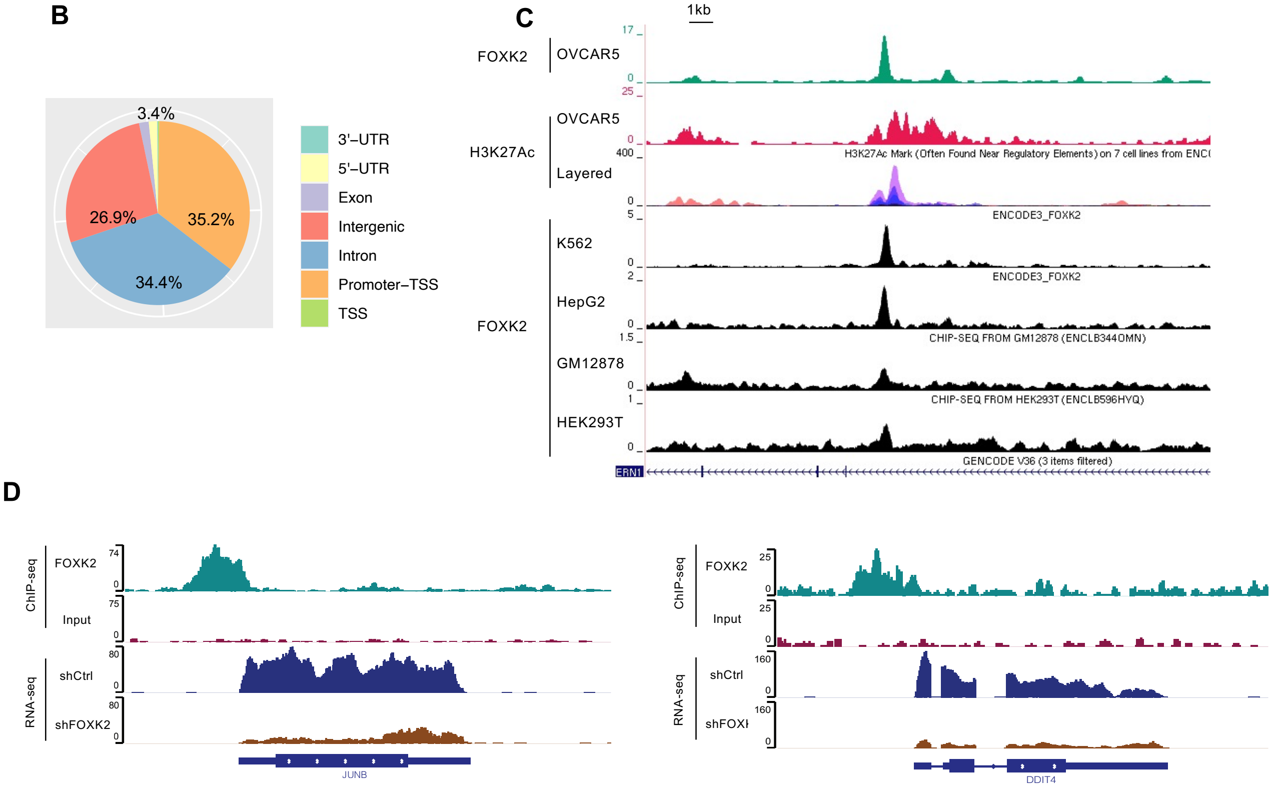

Supplementary Figure 5. FOXK2 directly regulates IRE1α expression

A, Binding motif analysis of FOXK2 based on ChIP-seq peaks in OVCAR5 cells. **B,** Gene component analysis shows percent distribution of FOXK2 binding sites measured by ChIP-seq in OVCAR5 cells. **C,** Track views of FOXK2 ChIP-seq peaks and H3K27Ac ChIP-seq peaks in OVCAR5 cells, overlayed H3K27Ac enrichment from ENCODE in 7 different cell lines (GM12878, H1-hESC, HSMM, HUVEC, K562, NHEK, NHLF), and FOXK2 ChIP-seq peaks in K562, HepG2, GM12878, HEK293T cell lines from ENCODE at the *ERN1* gene. **D,** Track views of FOXK2 ChIP-seq peaks in OVCAR5 cells, and RNA-seq in shFOXK2 transfected OVCAR5 cells at genes involved in UPR (*JUNB* and *DDIT4*).

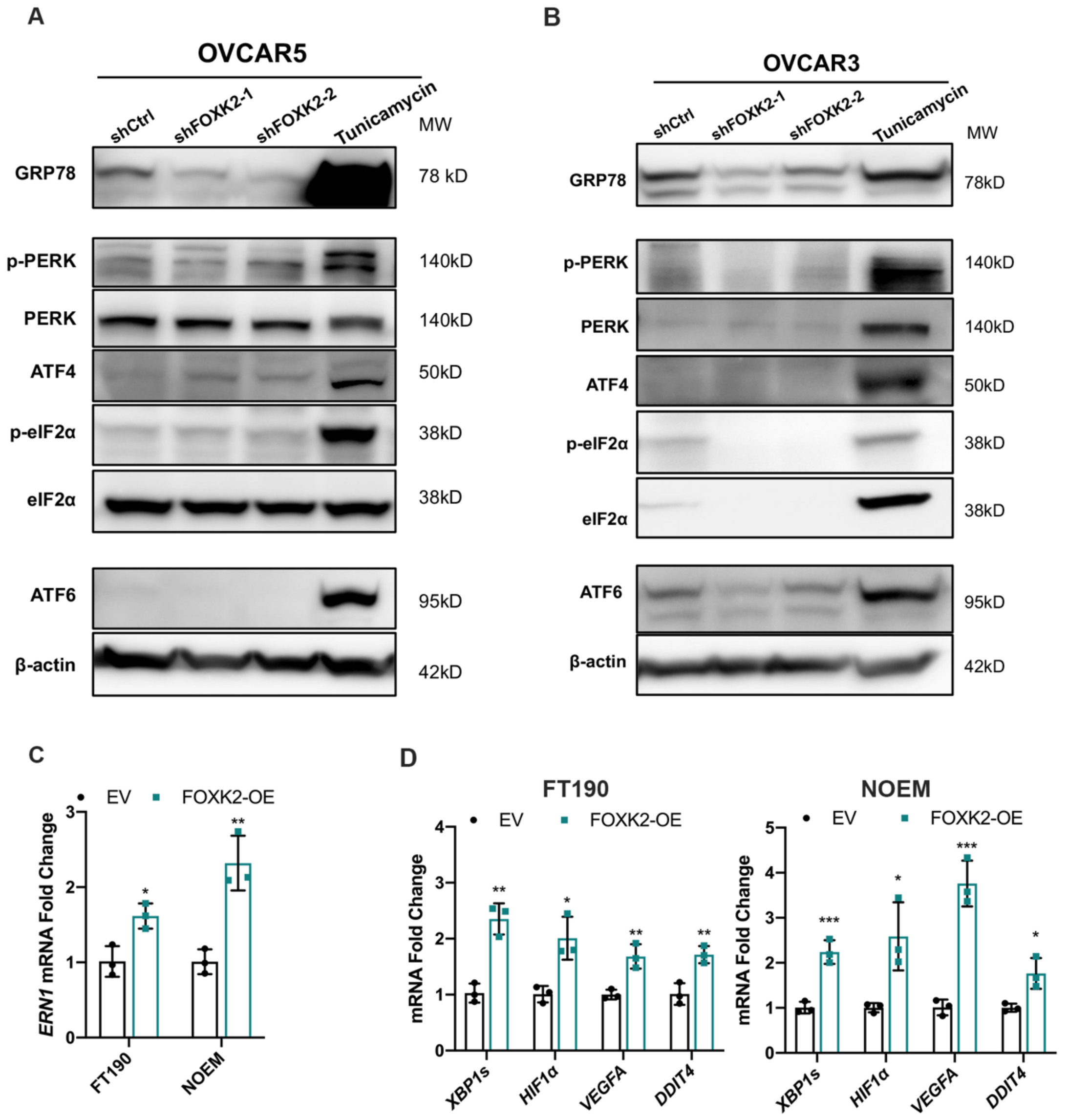

Supplementary Figure 6. Effects of FOXK2 on UPR signaling. A-B, Western blot measures GRP78, p-PERK, PERK, ATF4, p-eIF2α, eIF2α, ATF6 and β-actin in OVCAR5 (**A**) and OVCAR3 (**B**) cells transduced with shCtrl or shFOXK2 (n = 3). Treatment with 2.5μg/mL tunicamycin was used as a positive control (n = 3). **C,** mRNA expression levels (n = 3) of *ERN1* in FT190 and NOEM cells transduced with EV or FOXK2-OE. **D,** qRT-PCR measures mRNA expression levels of *XBP1s, HIF1α, VEGFA* and *DDIT4* in FT190 and NOEM cells transduced with EV or FOXK2 (n=3 replicates). For all panels: * p<0.05, ** p<0.01, *** p<0.005. Data were analyzed by unpaired two-tailed Student's t test.

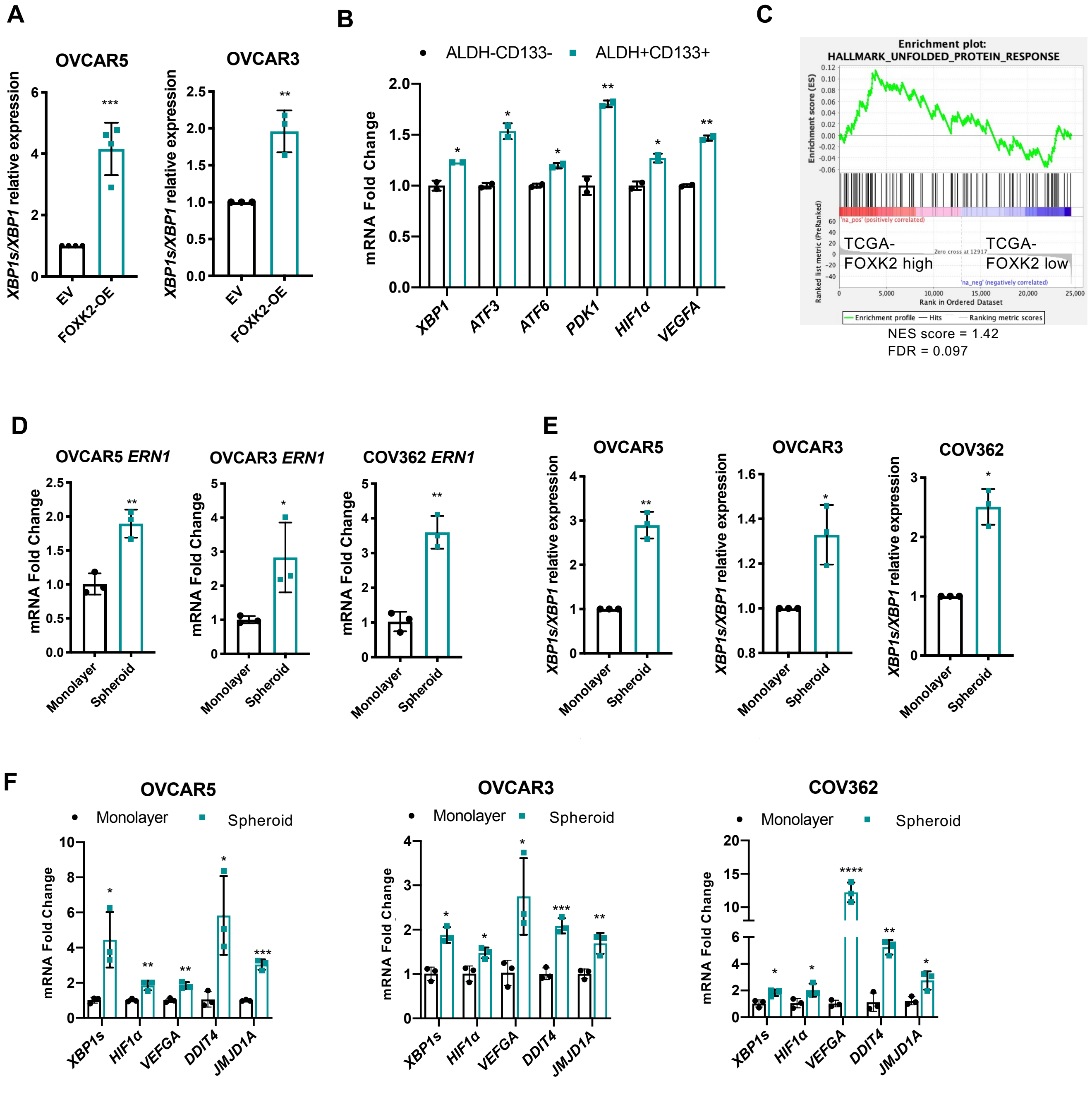

A
OVCAR5
OVCAR3
XBP1s/XBP1 relative expression
EV
FOXK2-OE
B
ALDH-CD133-
ALDH+CD133+
mRNA Fold Change
XBP1
ATF3
ATF6
PDK1
HIF1α
VEGFA
C
Enrichment plot:
HALLMARK_UNFOLDED_PROTEIN_RESPONSE
Enrichment score (ES)
Ranked list metric (PreRanked)
TCGA-
FOXK2 high
TCGA-
FOXK2 low
Zero cross at 12917
Rank in Ordered Dataset
Enrichment profile
Hits
Ranking metric scores
NES score = 1.42
FDR = 0.097
D
OVCAR5 ERN1
OVCAR3 ERN1
COV362 ERN1
Monolayer
Spheroid
E
OVCAR5
OVCAR3
COV362
F
OVCAR5
OVCAR3
COV362
Monolayer
Spheroid
XBP1s
HIF1α
VEFGA
DDIT4
JMJD1A

Supplementary Figure 7. FOXK2 upregulates IRE1α expression

A, Ratios measured by qRT-PCR (n = 3) of the *XBP1* mRNA spliced isoform (*XBP1s*) relative to the unspliced *XBP1* (*XBP1u*) in OVCAR5 and OVCAR3 cell lines transduced with EV or FOXK2. **B,** mRNA levels of *XBP1, ATF3, ATF6, VEGFA, PDK1,* and *HIF1α* determined by RNA-seq in ALDH-/CD133- and ALDH+/CD133+ FACS-sorted cells from OVCAR5 cells (n = 2). **C,** GSEA enrichment plot of "*Hallmark unfolded protein response*" gene set based on analysis of human ovarian tumors with high versus low *FOXK2* expression from TCGA (n = 427). **D-E,** *ERN1* mRNA levels (**D**) and ratios of spliced *XBP1*/unspliced *XBP1* mRNA (*XBP1s/XBP1*) (**E**) measured by qRT-PCR in OVCAR5, OVCAR3 and COV362 cells cultured as monolayers or as ALDH^{+}-enriched spheroids (n = 3). **F,** qRT-PCR measures mRNA expression levels (n = 3) of *XBP1s, HIF1α, VEGFA, DDIT4* and *JMJD1A* in OVCAR5, OVCAR3, and COV362 OC cells cultured as monolayer or as ALDH^{+}-enriched spheroids. For all panels: * p<0.05, ** p<0.01, *** p<0.005, **** p<0.0001. Data were analyzed by unpaired two-tailed Student's t test.

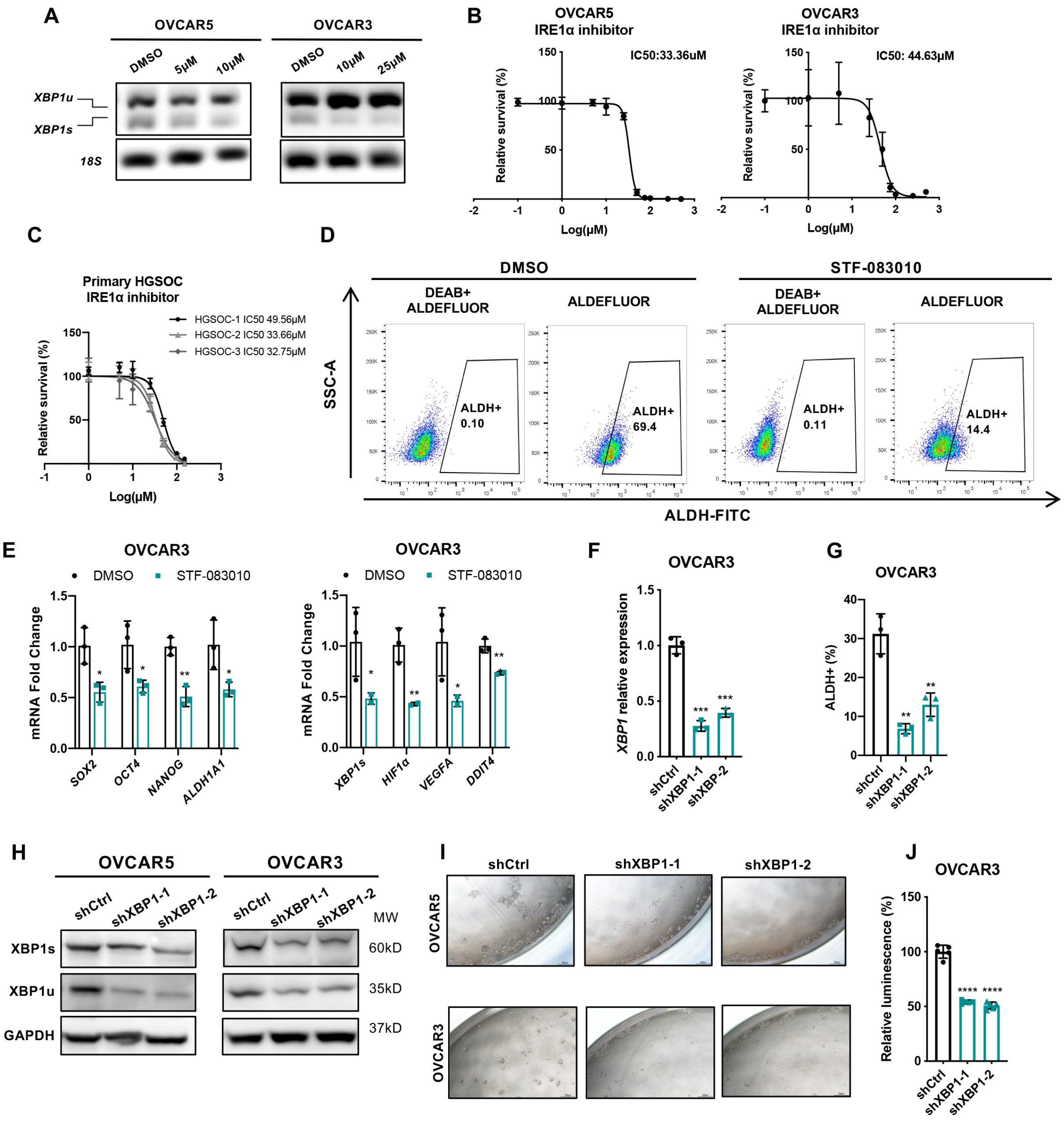
A
OVCAR5
OVCAR3
DMSO
5μM
10μM
DMSO
10μM
25μM
XBP1u
XBP1s
18S
B
OVCAR5
IRE1α inhibitor
IC50:33.36uM
OVCAR3
IRE1α inhibitor
IC50: 44.63μM
Relative survival (%)
Log(μM)
C
Primary HGSOC
IRE1α inhibitor
HGSOC-1 IC50 49.56μM
HGSOC-2 IC50 33.66μM
HGSOC-3 IC50 32.75μM
Relative survival (%)
Log(μM)
D
DMSO
STF-083010
DEAB+
ALDEFLUOR
ALDEFLUOR
SSC-A
ALDH+
0.10
ALDH+
69.4
ALDH+
0.11
ALDH+
14.4
ALDH-FITC
E
OVCAR3
DMSO
STF-083010
mRNA Fold Change
SOX2
OCT4
NANOG
ALDH1A1
XBP1s
HIF1α
VEGFA
DDIT4
F
OVCAR3
XBP1 relative expression
shCtrl
shXBP1-1
shXBP-2
G
OVCAR3
ALDH+ (%)
shCtrl
shXBP1-1
shXBP1-2
H
OVCAR5
OVCAR3
shCtrl
shXBP1-1
shXBP1-2
MW
XBP1s
XBP1u
GAPDH
60kD
35kD
37kD
I
shCtrl
shXBP1-1
shXBP1-2
OVCAR5
OVCAR3
J
OVCAR3
Relative luminescence (%)
shCtrl
shXBP1-1
shXBP1-2

Supplementary Figure 8. Inhibition of IRE1α/XBP1 blocks stemness features

A, A *XBP1* splicing assay shows effects of treatment with the IRE1α inhibitor STF-083010 on *XBP1s* and *XBP1u* mRNA levels in OVCAR5 and OVCAR3 cells. **B,** Half maximal inhibitory concentration (IC50) of STF-083010 on viability of OVCAR5 and OVCAR3 cells (n = 5 per dose). **C,** IC50 of STF-083010 on viability of cancer cells isolated from 3 HGSOC tumors and cultured in monolayer conditions (n = 5 per dose). **D,** Flow cytometry analysis shows percent ALDH+ population in OVCAR5 cells treated with STF-083010 or DMSO (n = 3). **E,** qRT-PCR measures mRNA levels of *SOX2, OCT4*, *NANOG* and *ALDH1A1* (left) and *XBP1s, HIF1α, VEGFA,* and *DDIT4* (right) in OVCAR3 cells treated with STF-083010 or DMSO (n = 3). **F,** *XBP1* mRNA levels in OVCAR3 cells transduced with shRNAs directed at *XBP1* (shXBP1-1 and shXBP1-2) or non-targeting shRNA (shCtrl) (n = 3). **G,** ALDH+ CSCs population in OVCAR3 cells transfected with shXBP1 or shCtrl (n = 3) measured by flow cytometry. **H,** Western-blot analysis of XBP1s and XBP1u in shXBP1 and shCtrl transduced OVCAR5 and OVCAR3 cells (n = 3). **I,** Pictures of spheroids formed from shXBP1 and shCtrl transduced OVCAR5 and OVCAR3 cells (n = 5) (magnification: 20X). **J,** Quantitative analysis of cell viability measures spheroid formation from OVCAR3 cells transduced with shXBP1 or shCtrl (n = 5). For all panels: * $p<0.05$, ** $p<0.01$, *** $p<0.005$, **** $p<0.0001$. Data were analyzed by unpaired two-tailed Student's t test.

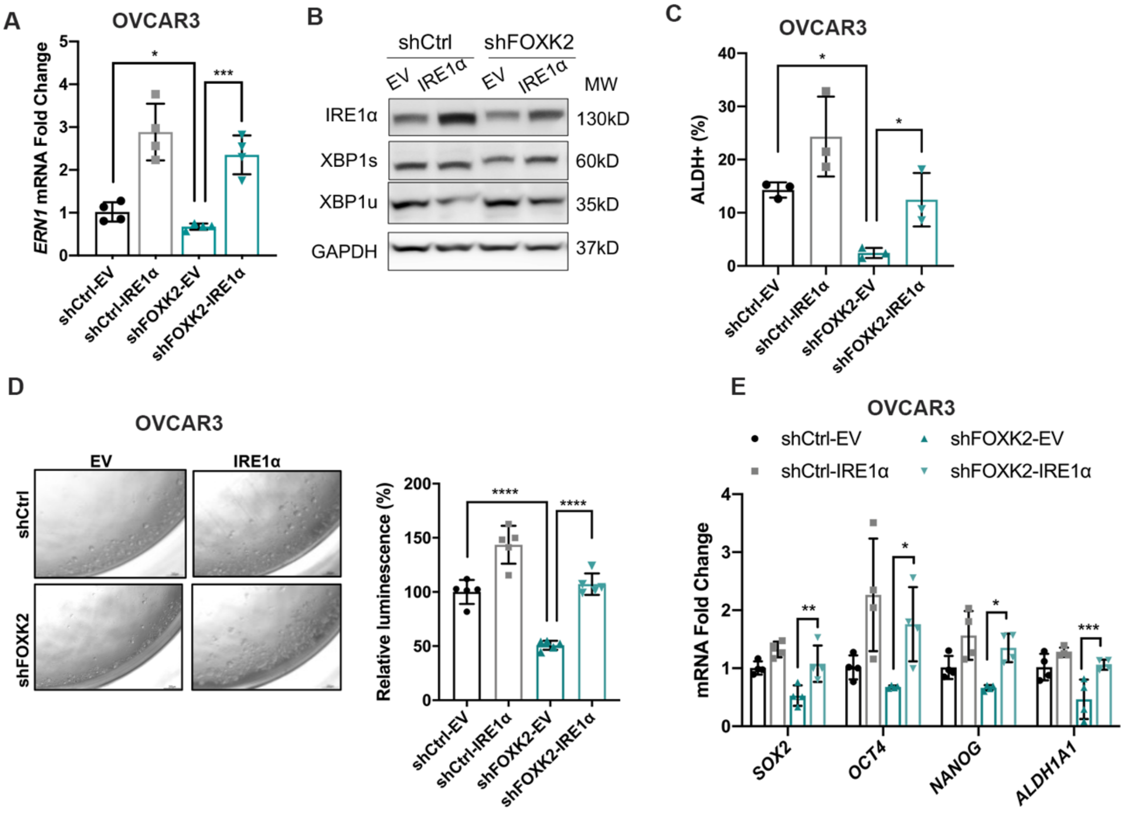

Supplementary Figure 9. Effects of restoring IRE1α in FOXK2-deficient cells

A, B, Expression levels of *ERN1* mRNA determined by qRT-PCR (n = 3) (**A**) and protein levels (western blotting) of IRE1α, spliced XBP1 (XBP1s), unspliced XBP1 (XBP1u) and GAPDH (loading control) (**B**) in shCtrl and shFOXK2 transduced OVCAR3 cells transfected with IRE1α-expressing vector (shCtrl-IRE1α or shFOXK2-IRE1α) or empty vector (shCtrl-EV or shFOXK2-EV). **C,** ALDH+ population measured by flow cytometry in shCtrl and shFOXK2 transduced OVCAR3 cells transfected with IRE1α or EV (n = 3). **D,** Pictures of spheroids (left, 20X magnification) and spheroid formation (n = 5) measured by cell viability (right) in shCtrl and shFOXK2 transduced OVCAR3 cells transfected with IRE1α or EV. **E,** mRNA expression levels of stemness genes (*SOX2, OCT4, NANOG* and *ALDH1A1*) in shCtrl and shFOXK2 transduced OVCAR5 cells transfected with IRE1α or EV. For all panels: * $p<0.05$, ** $p<0.01$, *** $p<0.005$, **** $p<0.0001$. Data were analyzed by two-way ANOVA with multiple comparisons when comparing more than two groups.

Supplementary Materials 2
Supplementary Tables

Supplementary Table 1. Estimated 1/stem cell frequency in shCtrl and shFOXK2 OVCAR5 cells in serial dilution assay experiment#

Group	Lower	Estimate	Upper
shCtrl	11533	5281	2418
shFOXK2	488060	67469	9327

# p = 0.0019; n = 12.

Supplementary Table 2. åEffects of FOXK2 on tumor initiation capacity#

Group	Dose	Tested	# Tumors
shCtrl	2,500	4	4
	5,000	4	1
	10,000	4	3
shFOXK2	2,500	4	0
	5,000	4	1
	10,000	4	0

Tumor initiation was assessed on day 11; n = 12.

Supplementary Table 3. Numbers of xenografts generated from serial dilutions of shCtrl and shFOXK2 cells transduced with EV or Foxk2#

Group	Dose	Tested	# Tumors
shCtrl-EV	5,000	4	4
	2,500	4	3
	1,000	4	1
shCtrl-Foxk2	5,000	4	4
	2,500	4	4
	1,000	4	4
shFOXK2-EV	5,000	4	1
	2,500	4	1
	1,000	4	0
shFOXK2-Foxk2	5,000	4	3
	2,500	4	2
	1,000	4	3

Tumor initiation was assessed on day 23; n = 12.

Supplementary Table 4. Estimated 1/stem cell frequency in shFOXK2 cells transduced with murine Foxk2 in the rescue serial dilution assay experiment#

Group	Lower	Estimate	Upper	P-value	Compare with
shCtrl-EV	4062	1858	850		
shCtrl-Foxk2	1236	1	1	0.00913	shCtrl-EV
shFOXK2-EV	60350	15039	3747	0.00377	shCtrl-EV
shFOXK2-Foxk2	5394	2449	1111	0.0105	shFOXK2-EV

Tumor initiation was assessed on day 23; n = 12.

Supplementary Table 5. Top 50 upregulated genes in OVCAR5 cells transduced with shRNA targeting *FOXK2* vs control shRNA (RNA-sequencing analysis).

hgnc_symbol	logFC	logCPM	P Value	FDR
HSP90AA1	4.42648695	12.3777237	2.97E-56	4.43E-53
KIF20B	5.00884785	7.31163683	3.87E-56	5.25E-53
MALAT1	5.05963275	12.4771226	3.93E-53	4.52E-50
CENPE	4.87151516	7.54914001	6.36E-50	6.79E-47
ESF1	4.96780741	6.695104	3.06E-48	2.69E-45
LRRCC1	5.83905133	5.2271597	7.74E-48	6.43E-45
CCDC88A	4.75001833	8.22017064	1.79E-46	1.34E-43
CCDC18	5.3793613	5.42790589	1.58E-45	9.81E-43
PPIG	4.65114674	7.87347465	9.22E-45	5.30E-42
CENPF	4.12143881	8.13946135	1.37E-43	7.07E-41
GOLGA4	4.25568933	7.30435529	2.32E-43	1.12E-40
ANKRD12	4.09286258	7.3087893	1.10E-40	4.84E-38
SMC4	4.24407453	8.92310203	3.65E-40	1.51E-37
SGO2	3.9606967	6.52787652	7.73E-38	2.57E-35
CLSPN	4.26133504	6.58670854	1.38E-37	4.39E-35
PHIP	4.22102839	6.98790334	1.61E-37	5.00E-35
ANKRD26	4.51475839	5.40175303	1.75E-37	5.33E-35
LYAR	3.99911	6.41514189	6.04E-36	1.77E-33
BRCA2	4.74872761	5.10262242	1.25E-34	3.39E-32
TOP2A	3.7996555	10.20815	1.57E-34	4.19E-32
UACA	3.98042917	8.14778654	6.63E-34	1.62E-31
ATRX	4.01994343	7.23796293	6.97E-34	1.68E-31
RIF1	3.87475973	6.98127815	8.68E-34	2.06E-31
HELLPAR	5.97231239	3.19172165	3.41E-33	7.83E-31
SMC2	3.8135413	7.12923393	1.83E-32	3.92E-30
NEXN	4.44312371	4.28420491	2.25E-32	4.73E-30
HMMR	3.36245044	7.29454819	9.82E-32	2.04E-29
ATAD5	4.27912516	5.47245048	5.03E-31	1.02E-28
UPF3B	3.9652371	5.52079399	8.58E-31	1.71E-28
CWF19L2	3.82309259	5.25074793	2.60E-30	5.04E-28
ZC3H15	3.20150904	7.65091706	2.96E-30	5.59E-28
RBM25	3.69091814	8.49137703	4.39E-30	8.19E-28
NOP58	3.21116651	7.79671732	1.10E-29	1.96E-27
NSRP1	4.18010982	6.00228856	1.57E-29	2.72E-27
CEP290	4.01572425	5.1771293	1.99E-29	3.41E-27
EEA1	4.14697109	5.17317279	2.01E-29	3.41E-27
ANKRD36C	4.23199653	5.41016305	2.76E-29	4.63E-27
ASPM	3.50157779	6.44020905	5.51E-29	9.05E-27
CSPP1	3.99603195	5.98397334	7.19E-29	1.16E-26
ZFPM2	5.48777242	3.63400198	8.63E-29	1.37E-26
DNAJC2	3.39072386	6.03191648	5.60E-28	8.46E-26
ANKRD36	4.18876955	3.8595243	6.85E-28	1.02E-25
CIP2A	3.64555478	5.77738321	1.20E-27	1.75E-25
NEMF	3.97240207	6.57490038	1.40E-27	2.02E-25
FAM133B	4.14859253	4.65437557	2.35E-27	3.31E-25
BDP1	3.8740607	5.93602549	3.47E-27	4.71E-25
AKAP9	3.59262427	6.31086781	3.58E-27	4.82E-25
SMC6	3.50202102	6.86847796	4.37E-27	5.83E-25
MNS1	4.61044452	3.71791153	7.29E-27	9.64E-25
CCDC112	3.69046998	4.50503905	9.55E-27	1.25E-24

Supplementary Table 6. Top 50 downregulated genes in OVCAR5 cells transduced with shRNA targeting *FOXK2* vs control shRNA (RNA-sequencing analysis).

hgnc_symbol	logFC	logCPM	P Value	FDR
SAA2	-9.3452095	6.36735934	8.10E-88	1.21E-83
CCL2	-7.5137551	6.17005748	1.24E-75	9.28E-72
C15orf48	-9.5564094	5.26398902	1.21E-69	4.41E-66
CXCL6	-9.8760962	5.04578526	1.48E-69	4.41E-66
CXCL1	-7.4552614	6.26413984	1.22E-67	3.03E-64
SPINT1	-5.2904781	7.3258643	4.46E-63	9.52E-60
SAA1	-7.845524	8.17045018	7.59E-63	1.42E-59
ICAM1	-5.3480143	8.14323745	9.83E-61	1.63E-57
FN1	-5.0059352	8.84708553	2.33E-55	2.90E-52
SOD2	-4.4515651	9.0345816	1.09E-49	1.08E-46
STRA6	-5.8991971	4.88685172	2.93E-49	2.74E-46
LCN2	-5.087346	8.87316865	4.77E-47	3.75E-44
CXCL5	-9.253319	3.56850349	2.35E-46	1.67E-43
SLC2A3	-5.0373596	5.71333662	4.67E-46	3.17E-43
PTGS2	-6.8383657	4.94900363	7.03E-46	4.57E-43
MIR3142HG	-9.259933	3.57591313	5.83E-45	3.48E-42
C1S	-5.0961584	5.199625	2.14E-44	1.19E-41
WNT10A	-6.9225075	3.97782849	4.14E-44	2.21E-41
CXCL2	-5.3888752	5.01399776	1.49E-43	7.41E-41
C1R	-5.1227135	4.87703283	4.14E-43	1.93E-40
DDR1	-3.7889322	8.8459918	9.65E-43	4.37E-40
IL4I1	-5.6933207	4.19248583	3.28E-40	1.40E-37
PLAT	-4.6071468	9.05850559	1.00E-39	4.04E-37
EHF	-7.409947	4.4756253	1.31E-39	5.16E-37
CXCL3	-5.7230885	4.05039888	1.08E-38	4.13E-36
LTB	-6.0627835	4.08513389	1.32E-38	4.95E-36
MMP24	-4.4699712	6.46932681	1.55E-38	5.65E-36
IL6	-6.8177296	3.5174896	3.18E-38	1.13E-35
MMP7	-6.3047826	3.56395913	4.67E-38	1.62E-35
ZC3H12A	-4.1674132	6.308499	7.34E-38	2.49E-35
NEURL3	-6.2784065	3.69601053	9.51E-38	3.09E-35
PIGR	-6.0412273	3.45134287	2.17E-36	6.48E-34
TNS3	-3.4275645	9.41966662	1.36E-35	3.90E-33
CXCL8	-7.0556165	5.73574888	6.25E-35	1.76E-32
S100A9	-7.7126997	2.89997063	7.09E-35	1.96E-32
TNFRSF9	-4.6938918	5.09924569	1.94E-34	5.08E-32
JUP	-3.7095043	7.5986388	2.83E-34	7.30E-32
CX3CL1	-4.4143135	5.32328616	4.39E-34	1.11E-31
C3	-7.8751441	9.56369624	5.29E-34	1.32E-31
TP53INP2	-4.0330712	5.302806	1.70E-33	3.96E-31
SYNPO	-3.7921391	6.1085625	5.26E-33	1.18E-30
IRAK2	-3.8700677	6.03794344	5.31E-33	1.18E-30
TNIP1	-3.276983	8.82382288	6.85E-33	1.50E-30
MX1	-3.6340139	6.18866912	1.71E-30	3.37E-28
CREB3L1	-4.1116025	5.54923684	4.87E-30	8.99E-28
GDF15	-3.5108614	6.30107313	9.57E-30	1.72E-27
SRGN	-5.0721989	3.16242457	1.56E-29	2.72E-27
TMPRSS2	-5.8256411	2.7505314	4.82E-29	7.99E-27
TRIM29	-4.009073	4.47948753	6.56E-29	1.07E-26
CSF2	-5.9736616	3.89882907	1.15E-28	1.81E-26

Supplementary Table 7. Top 50 FOXK2 target genes downregulated in OVCAR5 cells transduced with shRNA targeting *FOXK2* vs control shRNA (ChIP-seq and RNA-seq analyses).

hgnc_symbol	logFC	PValue	FDR	log10.Pvalue
CDH5	-8.5992822	3.96E-16	1.43E-14	15.4023048
CXCL8	-7.0556165	6.25E-35	1.76E-32	34.20412
RORC	-5.1523636	1.92E-24	1.94E-22	23.7166988
LRMDA	-4.9020771	2.80E-16	1.03E-14	15.552842
PIK3IP1	-4.4826597	4.09E-21	2.91E-19	20.3882767
SOD2	-4.4515651	1.09E-49	1.08E-46	48.9625735
TCP11L2	-4.3193458	1.65E-18	8.32E-17	17.7825161
LBH	-4.131982	6.86E-25	7.32E-23	24.1636759
SPINT1-AS1	-4.0768094	6.92E-12	1.16E-10	11.1598939
CTSF	-3.9774153	6.02E-23	5.38E-21	22.2204035
SYNPO	-3.7921391	5.26E-33	1.18E-30	32.2790143
YPEL2	-3.7267337	4.88E-20	2.95E-18	19.3115802
TNIP1	-3.276983	6.85E-33	1.50E-30	32.1643094
ABHD4	-3.1997159	1.21E-23	1.16E-21	22.9172146
FHDC1	-3.1981169	1.11E-15	3.68E-14	14.954677
OTUD1	-3.1651045	5.41E-20	3.24E-18	19.2668027
PDK2	-3.119585	6.85E-20	4.03E-18	19.1643094
TMEM140	-2.9981038	1.70E-18	8.56E-17	17.7695511
ZNRF2P2	-2.8599748	4.21E-06	2.22E-05	5.3757179
GPR146	-2.7668206	6.48E-09	6.17E-08	8.18842499
NNMT	-2.7449146	2.81E-13	6.07E-12	12.5512937
MMD	-2.671993	1.16E-16	4.58E-15	15.935542
FBXO32	-2.5296542	1.25E-14	3.42E-13	13.90309
LRP10	-2.4824631	3.01E-20	1.87E-18	19.5214335
BIRC3	-2.4210472	2.11E-08	1.81E-07	7.67571754
HBP1	-2.400446	4.41E-12	7.71E-11	11.3555614
KLF9	-2.3952589	8.25E-08	6.22E-07	7.08354605
SDK1	-2.3657065	2.02E-14	5.35E-13	13.6946486
SLC35E2B	-2.3523009	4.01E-15	1.21E-13	14.3968556
MAML3	-2.265541	1.29E-09	1.41E-08	8.88941029
PDCD4-AS1	-2.2337891	8.68E-06	4.28E-05	5.06148027
TMEM45B	-2.2056702	7.72E-09	7.25E-08	8.1123827
TMEM45B	-2.2056702	7.72E-09	7.25E-08	8.1123827
HIVEP2	-2.2041039	4.30E-11	6.18E-10	10.3665315
LIPG	-2.1833576	9.85E-09	9.03E-08	8.00656377
CREBL2	-2.1718365	4.21E-11	6.07E-10	10.3757179
TBC1D14	-2.1691391	2.77E-12	5.07E-11	11.5575202
ELFN2	-2.1636516	2.66E-10	3.29E-09	9.57511836
NDRG1	-2.117665	1.16E-12	2.25E-11	11.935542
TCP11L1	-2.1103289	8.75E-10	9.85E-09	9.05799195
IGF1R	-2.1081727	1.75E-14	4.67E-13	13.756962
MAN1A1	-2.1060373	1.97E-07	1.38E-06	6.70553377
WIPI1	-2.0967221	2.26E-09	2.36E-08	8.64589156
TBC1D9	-2.0909024	2.01E-10	2.53E-09	9.69680394
ABCA1	-2.0717651	2.80E-08	2.34E-07	7.55284197
SIDT2	-2.0529082	4.90E-12	8.50E-11	11.3098039
SELENOP	-2.0460248	5.63E-06	2.88E-05	5.24949161
YPEL3	-2.0376441	2.34E-06	1.30E-05	5.63078414
ERN1	-2.0312542	6.67E-11	9.15E-10	10.1758742
PDP1	-2.0151969	1.80E-10	2.29E-09	9.74472749

Supplementary Table 8. Analysis of FOXK2 ChIP-seq binding peak in the E*RN1* gene

**Peak score**	31.8
**Peak location**	intron 2 of 21
**Distance to TSS**	47839
**Log2 FC**	2.54964000
**Target vs Input p value**	1.24E-09
**Target vs Input adjusted p value**	2.00E-07

Supplementary Table 9. Estimated stem cell frequency of shFOXK2 cells with IRE1α rescue expression in serial dilution assay#

Group	Dose	Tested	# tumors
shCtrl-EV	5,000	4	2
	2,500	4	3
	1,000	4	0
shCtrl-IRE1α	5,000	4	3
	2,500	4	4
	1,000	4	1
shFOXK2-EV	5,000	4	1
	2,500	4	0
	1,000	4	0
shFOXK2-IRE1α	5,000	4	3
	2,500	4	4
	1,000	4	0

Tumor initiation was assessed on day 21; n = 12.

Supplementary Table 10. Estimated stem cell frequency of shFOXK2 cells with IRE1α rescue expression in serial dilution assay#

Group	Lower	Estimate	Upper	P-value	Compare with
shCtrl-EV	11898	4915	1949		
shCtrl-IRE1α	4753	2171	992	0.179	shCtrl-EV
shFOXK2-EV	21935	31434	4505	0.0451	shCtrl-EV
shFOXK2-IRE1	5855	2641	1191	0.00375	shFOXK2-EV

Tumor initiation was assessed on day 21; n = 12.

Supplementary Table 11. Cell culture conditions

Cell line	Culture medium
OVCAR5	RPMI 1640 (Corning), 10% fetal bovine serum (FBS) (Corning), 1% penicillin/streptomycin (HyClone), and 1X GlutaMAX (Gibco).
OVCAR3	RPMI 1640 (ATCC), 20% FBS, 1% penicillin/streptomycin and 0.01 mg/mL recombinant human insulin (Gibco).
COV362	Dulbecco's Modified Eagle Medium (DMEM) (Corning), 10% FBS, 1% penicillin/streptomycin, 1X GlutaMAX.
OVCAR4, Kuramochi	RPMI 1640, 10% FBS, 1% penicillin/streptomycin.
NoEM, HeyA8, OVCAR8, CAOV3	DMEM, 10% FBS, 1% penicillin/streptomycin.
FT190, OV90	1:1 combination of MCDB 105 (Sigma Aldrich) and Medium 199 (Corning), 10% FBS and 1% penicillin/streptomycin

Supplementary Table 12. shRNA sequences

Gene	shRNA sequence
shFOXK2-1	5’-CCGGCCAGCCTCTGAAAGCAAATTACTCGAGTAATTTGCTTTCAGAGGCTGGTTTTT-3’
shFOXK2-2	5’-CCGGGCTGGCCTTAACACTCCTTAACTCGAGTTAAGGAGTGTTAAGGCCAGCTTTTT-3’
shXBP1-1	5’- CCGGGCCTGTCTGTACTTCATTCAACTCGAGTTGAATGAAGTACAGACAGGCTTTTT -3’
shXBP1-2	5’- CCGGAGATCGAAAGAAGGCTCGAATCTCGAGATTCGAGCCTTCTTTCGATCTTTTTT-3’

Supplementary Table 13. qRT-PCR Primers

Gene	Forward Primer (5' to 3')	Reverse Primer (5' to 3')	Ref.
FOXK2	TCAAAGCCGCCTTACTCCTA	TGGGTCTATCCTCCAGAACG	
18S	CGTCTGCCCTATCAACTTTC	GATGTGGTAGCC GTTTCTC	
SOX2	TGCTGCCTCTTTAAGACTAGGAC	CCTGGGGCTCAAACTTCTCT	
OCT4	CTTCGCAAGCCTCATTTC	GAGAAGGCGAAATCCGAAG	
NANOG	AGATGCCTCACACGGAGACT	TTTGCGACACTCTTCTCTGC	
ALDH1A1	AGGGGCAGCCATTTCTTCTCA	CACGGGCCTCCTCCACATT	
ERN1	AGCAAGCTGACGCCCACTCTG	TGGGGCCCTTCCAGCAAAGGA	[1]
XBP1	GAAGCCAAGGGGAATGAAGT	GCTGGCAGGCTCTGGGGAAG	[1]
XBP1s	TGCTGAGTCCGCAGCAGGTG	GCTGGCAGGCTCTGGGGAAG	[1]
XBP1-SA	CCTGGTTGCTGAAGAGGAGG	CCATGGGGAGTTCTGGAG	[2]
HIF1α	ACGTTCCTTCGATCAGTTGTCACC	GGCAGTGGTAGTGGTGGCATTAG	
VEGFA	AATGTGAATGCAGACCAAAG	GACTTATACCGGATTTCTTC	
DDIT4	CATCAGGTTGGCACACAAGT	CCTGGAGAGCTCGGACTG	[3]
JMJD1A	TCAGGTGACTTTCGTTCAGC	CACCGACGTTACCAAGAAGG	[3]

Supplementary Table 14. Antibodies

Antibodies	Source	Cat#
Anti-FOXK2 (immunoblot)	Cell Signaling Technology	12008
Anti-FOXK2 (ILF1) (immunohistochemistry)	Abcam	ab84761
Anti-FOXK2 (ChIP and ChIP-seq)	Bethyl Lab	A301-729A
Anti-IRE1α	Cell Signaling Technology	3294
Anti-XBP1	Abcam	ab220783
Anti-XBP1s (D2C1F)	Cell Signaling Technology	12782
Anti-Histone H3 (acetyl K27)	Abcam	ab4729
Anti-GAPDH	Meridian	H86045M
Anti-α tubulin	Proteintech	66031-1-Ig
ATF4	Abcam	ab184909
ATF6	Cell Signaling Technology	65880S
eIF2α	Cell Signaling Technology	9722
Phospho-eIF2α	Cell Signaling Technology	3597
PERK	Cell Signaling Technology	3192
Phospho-PERK	Cell Signaling Technology	3179
BIP (GRP78)	Cell Signaling Technology	3177S

Supplementary Table 15. ChIP-PCR Primers

Gene	Forward Primer (5' to 3')	Reverse Primer (5' to 3')
FOXK2 ChIP ERN1	GATGCAAGAGACAGGAAATAAAG	CTTCAGGCCGGTGTTAAA
FOXK2 ChIP ERN1-control	GCAGAACGAAGAACCAGTC	GGCATCGGACAAGGTAATG
H3K27Ac ChIP ERN1	GATCTTTGCTGTACAGTGTTTG	TCAGGTGACCAGTGGATAA
H3K27Ac ChIP ERN1-control	AAGCCAATGTCACCAAGTT	TTCCCTCTACCTTTACCAGATA

Supplementary Table 16. CRISPR/dCas9 gRNA target sequences

gRNA	Target sequence
dCas9-ERN1-1	AGCAAAGTCAACATATGATA
dCas9-ERN1-2	TCCATCCAATTATTGGATGA
dCas9-ERN1-3	TTGTTTCAGTAGGATGAGTG

Supplementary References

1 Sustic, T. *et al.* A role for the unfolded protein response stress sensor ERN1 in regulating the response to MEK inhibitors in KRAS mutant colon cancers. *Genome Med* **10**, 90, doi:10.1186/s13073-018-0600-z (2018).

2 Song, M. *et al.* IRE1alpha-XBP1 controls T cell function in ovarian cancer by regulating mitochondrial activity. *Nature* **562**, 423-428, doi:10.1038/s41586-018-0597-x (2018).

3 Chen, X. *et al.* XBP1 promotes triple-negative breast cancer by controlling the HIF1alpha pathway. *Nature* **508**, 103-107, doi:10.1038/nature13119 (2014).
